# Supplementary material for: Heterocellular OSM-OSMR signalling reprograms fibroblasts to promote pancreatic cancer growth and metastasis
Source: Nat Commun. 2021 Dec 17;12:7336. doi: 10.1038/s41467-021-27607-8 (PMC8683436; doi:10.1038/s41467-021-27607-8)
Supplement: Supplementary file 1 — Supplementary Information [file 41467_2021_27607_MOESM1_ESM.pdf]

# Heterocellular OSM-OSMR signalling reprograms fibroblasts to promote pancreatic cancer growth and metastasis

Brian Y Lee[1*], Elizabeth K J Hogg[1*], Christopher R Below[1], Alexander Kononov[1], Adrian Blanco-Gomez[1], Felix Heider[1], Jingshu Xu[1], Colin Hutton[1], Xiaohong Zhang[1], Tamara Scheidt[2], Kenneth Beattie[3], Angela Lamarca[4,5], Mairéad McNamara[4,5], Juan W Valle[4,5], Claus Jørgensen[1#]

[1] Cancer Research UK Manchester Institute, The University of Manchester, Alderley Park, SK10 4TG, Manchester, United Kingdom

[2] Department of Molecular Biology, University of Salzburg, Salzburg, Austria

[3] FingerPrints Proteomics Facility, College of Life Sciences, University of Dundee, Dundee, DD1 5EH, United Kingdom

[4] Department of Medical Oncology, The Christie NHS Foundation Trust, Wilmslow Road, M20 4BX, Manchester, UK

[5] Institute of Cancer Sciences, University of Manchester, Wilmslow Road, M20 4BX, UK

* These authors contributed equally

[#] Corresponding author: Claus Jørgensen, claus.jorgensen@cruk.manchester.ac.uk

## SUPPLEMENTARY FIGURES:

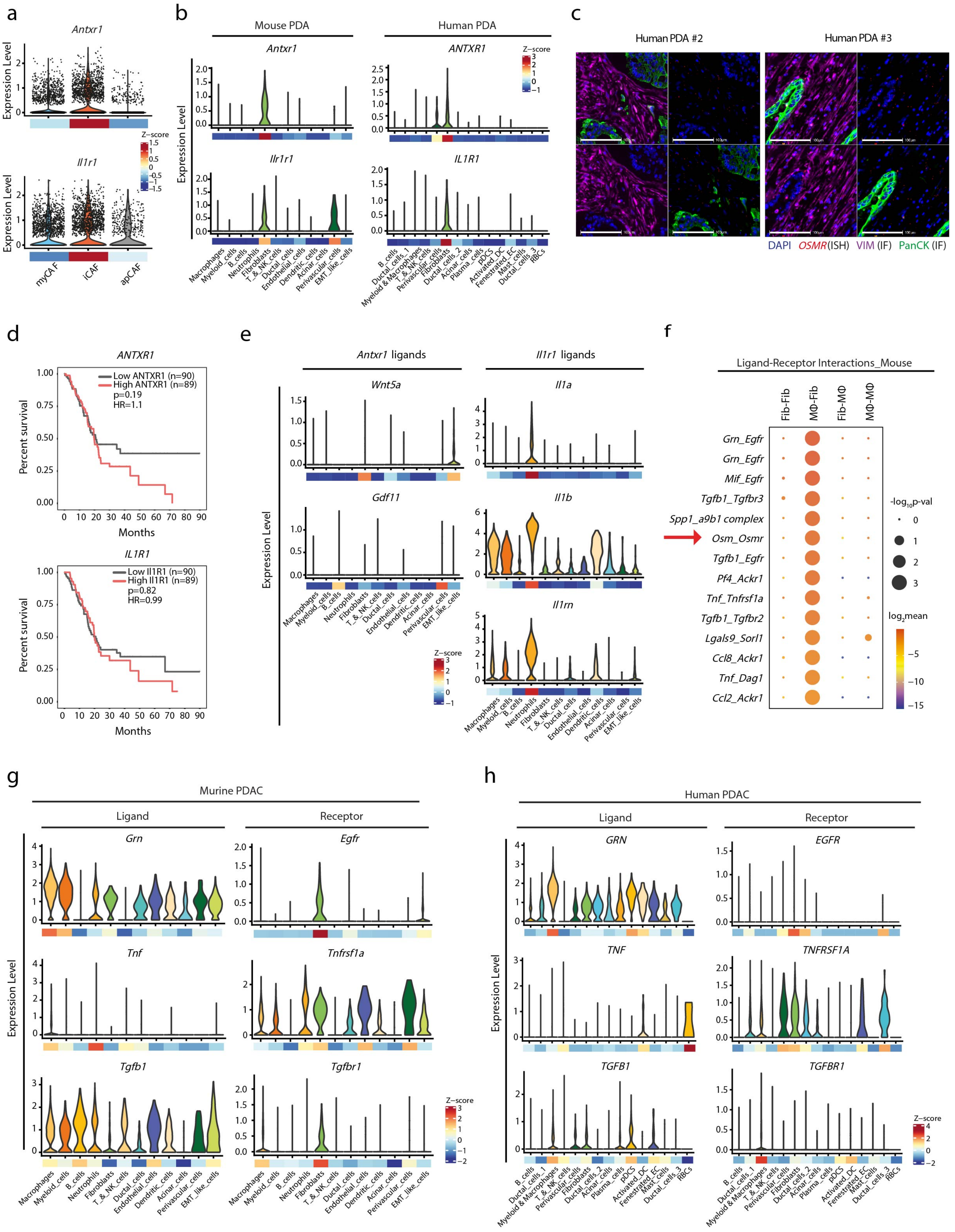
a
Antxr1
Il1r1
Expression Level
Z-score
myCAF
iCAF
apCAF
b
Mouse PDA
Human PDA
Antxr1
ANTXR1
Ilr1r1
IL1R1
Expression Level
Macrophages
Myeloid_cells
B_cells
Neutrophils
Fibroblasts
T_&_NK_cells
Ductal_cells
Endothelial_cells
Dendritic_cells
Acinar_cells
Perivascular_cells
EMT_like_cells
B_cells
Ductal_cells_1
Myeloid & Macrophages
T_&_NK_cells
Perivascular_cells
Fibroblasts
Ductal_cells_2
Acinar_cells
Plasma_cells
pDCs
Activated_DC
Fenestrated_EC
Mast_cells
Ductal_cells_3
RBCs
c
Human PDA #2
Human PDA #3
DAPI
OSMR(ISH)
VIM (IF)
PanCK (IF)
d
ANTXR1
Low ANTXR1 (n=90)
High ANTXR1 (n=89)
p=0.19
HR=1.1
Percent survival
Months
IL1R1
Low Il1R1 (n=90)
High Il1R1 (n=89)
p=0.82
HR=0.99
e
Antxr1 ligands
Il1r1 ligands
Wnt5a
Gdf11
Il1a
Il1b
Il1rn
Expression Level
f
Ligand-Receptor Interactions_Mouse
Fib-Fib
MΦ-Fib
Fib-MΦ
MΦ-MΦ
Grn_Egfr
Grn_Egfr
Mif_Egfr
Tgfb1_Tgfbr3
Spp1_a9b1 complex
Osm_Osmr
Tgfb1_Egfr
Pf4_Ackr1
Tnf_Tnfrsf1a
Tgfb1_Tgfbr2
Lgals9_Sorl1
Ccl8_Ackr1
Tnf_Dag1
Ccl2_Ackr1
-log10p-val
log2mean
g
Murine PDAC
Ligand
Receptor
Grn
Egfr
Tnf
Tnfrsf1a
Tgfb1
Tgfbr1
Expression Level
h
Human PDAC
Ligand
Receptor
GRN
EGFR
TNF
TNFRSF1A
TGFB1
TGFBR1
Expression Level

**Supplementary Figure 1. *OSMR* expression is associated with tumour-promoting inflammation and poor outcome in PDA**

**a)** Violin plots of normalised *Antxr1* and *Il1r1* expression in myCAF, iCAF and apCAFs in murine PDA with mean expression z-scores shown below (dataset: GSE129455). **b)** Violin plots of normalised expression levels of indicated receptors in different cell types of murine (left) and human (right) PDA. Mean expression z-scores shown below (datasets: GSE129455, phs001840.v1.p1). **c)** Representative in situ mRNA hybridisation (ISH) of *OSMR* multiplexed with immunofluorescence of VIM and PanCK in human resected PDA tumour (n=3, see Fig. 1d). 20X magnification. Upper left quadrant, full overlay; upper right, *OSMR*; lower left, *OSMR* with VIM; lower right, *OSMR* with PanCK. Scale bar=100 μm. **d)** Kaplan-Meier survival curves showing overall survival of 179 PDA patients from TCGA PanCancer PAAD dataset. Patients were stratified by high (Top 50%, n=89) and low (bottom 50%, n=90) *ANTXR1* (left) and *IL1R1* (right) expression. Analysis by log-rank test and cox-proportional hazard regression. HR: Hazard ratio. **e)** Violin plots of indicated ligand expression across individual cell types of murine PDA (dataset: GSE129455). **f)** Top 14 predicted interactions between murine macrophage-derived ligands and fibroblast-expressed receptors using CellPhoneDB receptor-ligand interaction statistical analysis (p-values <0.05). **g & h)** Violin plots of indicated ligand and receptor expression in individual cell types of murine (left) and human (right) PDA. Mean expression z-scores shown below (datasets: GSE129455, phs001840.v1.p1). Source data are provided as a Source Data file.

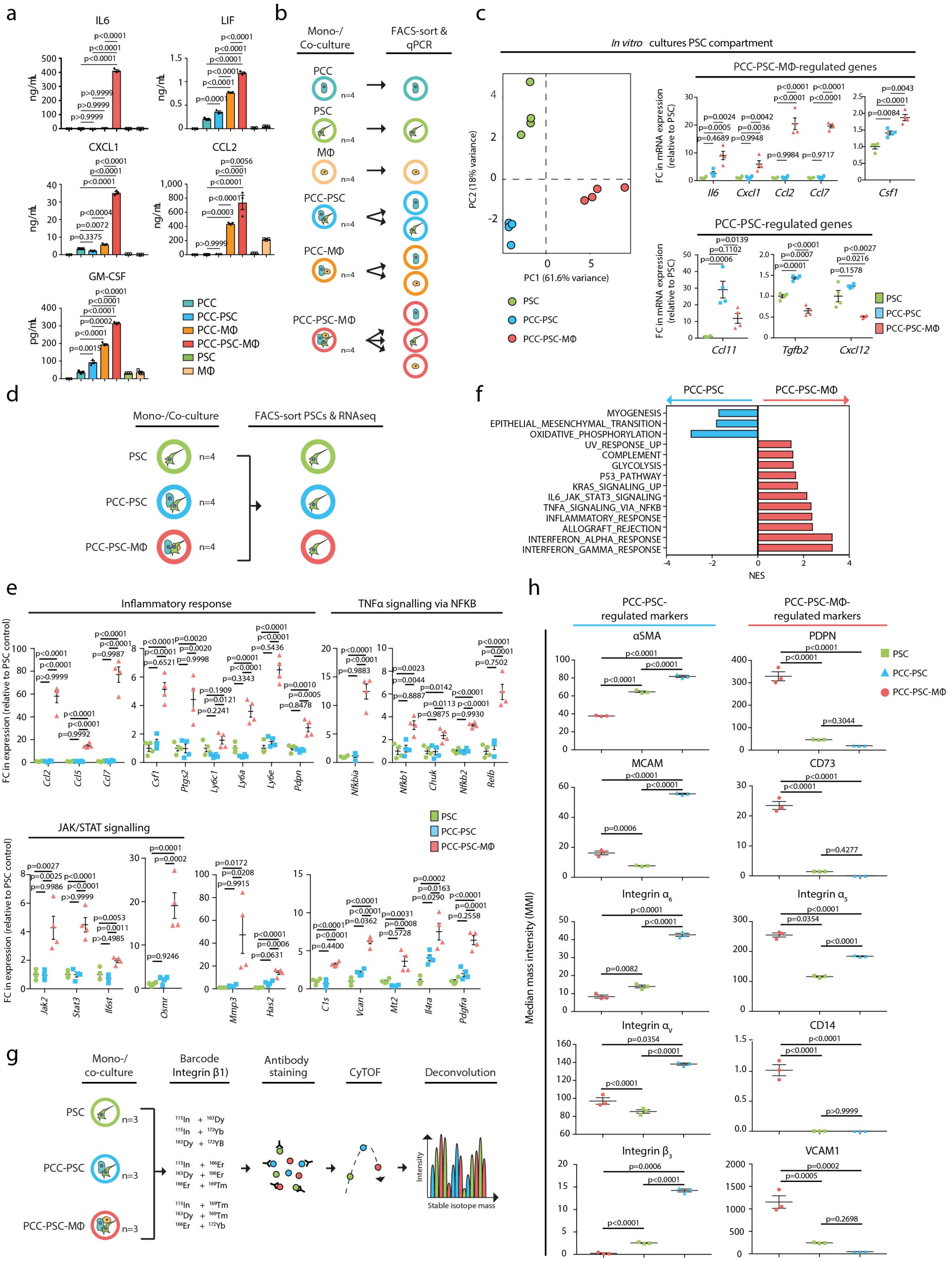

a
IL6
ng/mL
LIF
CXCL1
CCL2
GM-CSF
pg/mL
PCC
PCC-PSC
PCC-MΦ
PCC-PSC-MΦ
PSC
MΦ
b
Mono-/ Co-culture
FACS-sort & qPCR
PCC
PSC
MΦ
PCC-PSC
PCC-MΦ
PCC-PSC-MΦ
n=4
c
In vitro cultures PSC compartment
PC1 (61.6% variance)
PC2 (18% variance)
PSC
PCC-PSC
PCC-PSC-MΦ
PCC-PSC-MΦ-regulated genes
FC in mRNA expression (relative to PSC)
Il6
Cxcl1
Ccl2
Ccl7
Csf1
PCC-PSC-regulated genes
Ccl11
Tgfb2
Cxcl12
d
Mono-/Co-culture
FACS-sort PSCs & RNAseq
PSC
PCC-PSC
PCC-PSC-MΦ
n=4
f
PCC-PSC
PCC-PSC-MΦ
MYOGENESIS
EPITHELIAL_MESENCHYMAL_TRANSITION
OXIDATIVE_PHOSPHORYLATION
UV_RESPONSE_UP
COMPLEMENT
GLYCOLYSIS
P53_PATHWAY
KRAS_SIGNALING_UP
IL6_JAK_STAT3_SIGNALING
TNFA_SIGNALING_VIA_NFKB
INFLAMMATORY_RESPONSE
ALLOGRAFT_REJECTION
INTERFERON_ALPHA_RESPONSE
INTERFERON_GAMMA_RESPONSE
NES
e
Inflammatory response
TNFα signalling via NFKB
FC in expression (relative to PSC control)
Ccl2
Ccl5
Ccl7
Csf1
Ptgs2
Ly6c1
Ly6a
Ly6e
Pdpn
Nfkbia
Nfkb1
Chuk
Nfkb2
Relb
JAK/STAT signalling
Jak2
Stat3
Il6st
Osmr
Mmp3
Has2
C1s
Vcan
Mt2
Il4ra
Pdgfra
PSC
PCC-PSC
PCC-PSC-MΦ
g
Mono-/ co-culture
Barcode Integrin β1
Antibody staining
CyTOF
Deconvolution
PSC
PCC-PSC
PCC-PSC-MΦ
n=3
Intensity
Stable isotope mass
h
PCC-PSC-regulated markers
PCC-PSC-MΦ-regulated markers
Median mass intensity (MMI)
αSMA
PDPN
MCAM
CD73
Integrin α6
Integrin α5
Integrin αV
CD14
Integrin β3
VCAM1
PSC
PCC-PSC
PCC-PSC-MΦ

**Supplementary Figure 2. Macrophage–tumour cell interactions induce inflammatory fibroblasts in vitro**

**a)** Concentrations of selected soluble signals from mono- and co-cultures of PCC, PSC and MØ. Results displayed as mean ± SEM of 3 biological replicates, one-way ANOVA Tukey test. **b)** Experimental workflow. Selected genes were measured by RT-qPCR in FACS-isolated cells from mono- and co-cultures. **c)** Ligand mRNA expression in mono- and co-cultures of PCCs, PSCs and MØs (n=4 per culture condition) **LEFT:** Principle component analysis (PCA) plot. **RIGHT:** Fold change relative to PSC control. Results displayed as mean ± SEM, n=4, one-way ANOVA Tukey test. **d)** Experimental outline for isolation and RNAseq analysis of mCherry-labelled fibroblasts (PSC-mCherry) from mono- and co-cultures with PCC-EGFP or PCC-EGFP and MØ-TagBFP. Cells were cultured in mono- and co-cultures for 72 h. PSCs were FACS-isolated from each culture condition based on mCherry positivity and EGFP and TagBFP-negativity. **e)** Fold changes in normalised expression of indicated genes in fibroblasts from mono- and co-cultures (relative to PSC mono-culture). Results displayed as mean ± SEM, n=4. * $p<0.05$, ** $p<0.005$, *** $p<0.0001$, one-way ANOVA Tukey test. **f)** GSEA of Hallmark gene sets enriched in fibroblasts from PCC-PSC and PCC-PSC-MØ co-cultures. NES: normalised enriched score. **g)** Experimental workflow of mass cytometry-based analysis of fibroblasts in mono- and co-cultures. **h)** Mass cytometry marker analysis of PSCs in mono- and co-cultured fibroblasts with PCC and PCC-MØ. Median mass intensity (MMI) of indicated fibroblast marker expression levels are shown. Results displayed as mean ± SEM, n=3, one-way ANOVA Tukey test. Source data are provided as a Source Data file.

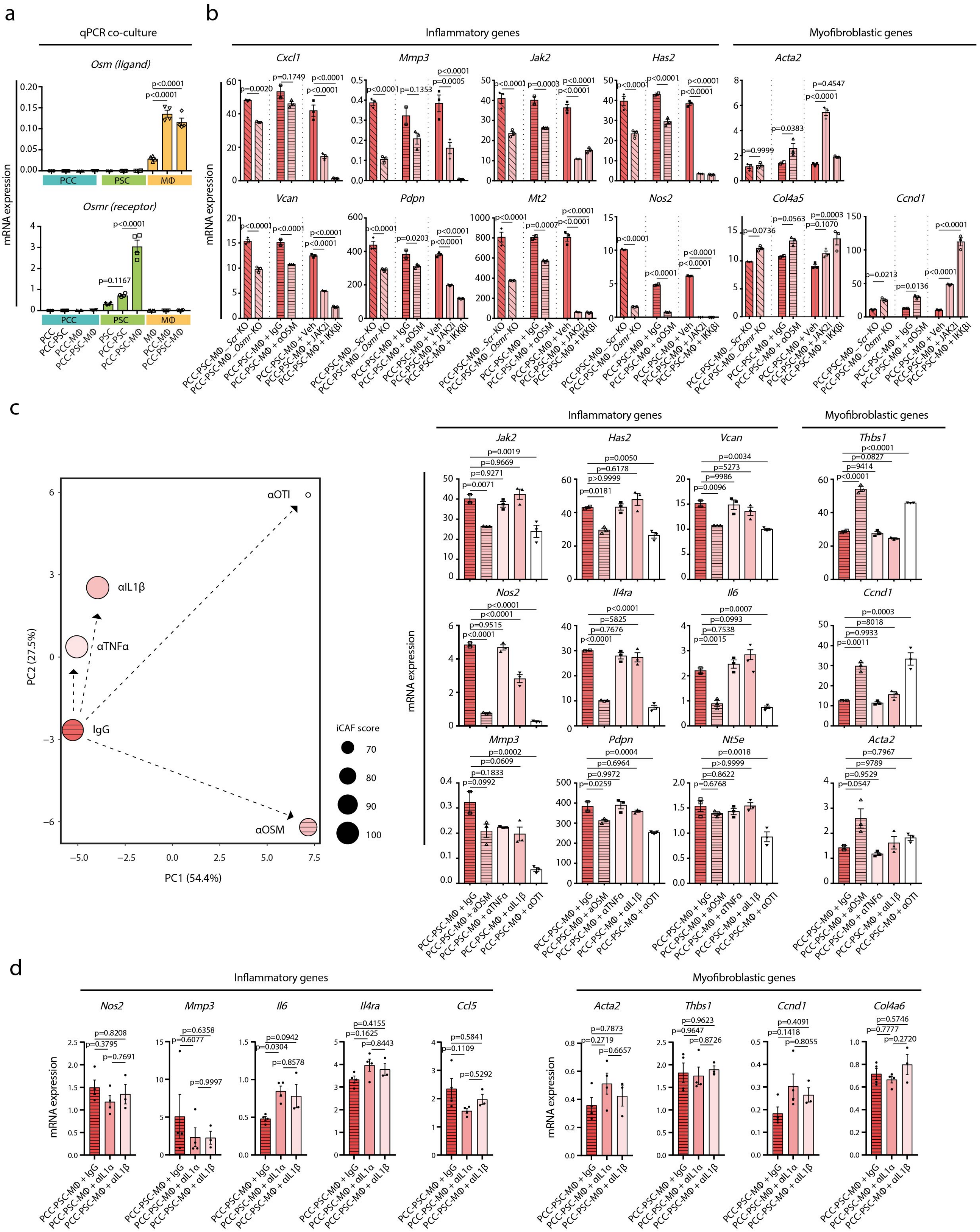
a
qPCR co-culture
Osm (ligand)
Osmr (receptor)
mRNA expression
PCC
PSC
MΦ
PCC-PSC
PCC-MΦ
PCC-PSC-MΦ
p<0.0001
p=0.1167
b
Inflammatory genes
Myofibroblastic genes
Cxcl1
Mmp3
Jak2
Has2
Acta2
Vcan
Pdpn
Mt2
Nos2
Col4a5
Ccnd1
PCC-PSC-MΦ_Scr-KO
PCC-PSC-MΦ_Osmr-KO
PCC-PSC-MΦ + IgG
PCC-PSC-MΦ + αOSM
PCC-PSC-MΦ + Veh
PCC-PSC-MΦ + JAK2i
PCC-PSC-MΦ + IKKβi
c
PC1 (54.4%)
PC2 (27.5%)
αOTI
αIL1β
αTNFα
IgG
αOSM
iCAF score
70
80
90
100
Thbs1
Il4ra
Il6
Nt5e
PCC-PSC-MΦ + IgG
PCC-PSC-MΦ + αOSM
PCC-PSC-MΦ + αTNFα
PCC-PSC-MΦ + αIL1β
PCC-PSC-MΦ + αOTI
d
Ccl5
Col4a6
PCC-PSC-MΦ + αIL1α
PCC-PSC-MΦ + αIL1β

**Supplementary Figure 3. Heterocellular OSM-OSMR signalling induces inflammatory fibroblasts**

**a)** Cell-type-specific mRNA expression of *Osm* and *Osmr* in mono- and co-cultures in vitro. Results displayed as mean ± SEM, n=4, one-way ANOVA Tukey test. **b)** RT-qPCR gene expression analysis of selected inflammatory and myofibroblastic genes in PSCs treated with pharmacological inhibitors or CRISPR-Cas9-medidated *Osmr* knockout (*Osmr*-KO) in fibroblasts. Results displayed as mean ± SEM, n=3, one-way ANOVA Tukey test. **c**) RT-qPCR gene expression analysis of selected inflammatory and myofibroblastic genes in PSCs treated with neutralising antibodies against OSM or TNFα or IL1β or combined OSM-TNFα-IL1β (OTI). PSCs were stimulated with PCC-PSC-MØ conditioned medium supplemented with control IgG antibody (2.5 ng/mL) or neutralising OSM (2.5 ng/mL, αOSM), TNFα (10 ng/mL, αTNFα), or IL1β (1 ng/mL, αIL1β) antibody, or combined neutralising OSM-TNFα-IL1β antibodies. **LEFT:** Change in expression state visualised by PCA plot across experimental conditions. iCAF score is a sum of mean z-values of 15 selected inflammatory genes. PC: principle component. **RIGHT:** Representative mRNA expression of inflammatory and myofibroblastic genes. Results displayed as mean ± SEM, n=3, one-way ANOVA Tukey test. **d)** As in (c), where PSCs were instead stimulated with PCC-PSC-MØ conditioned medium supplemented with control IgG antibody (3 µg/mL) or a neutralising IL1α (3 µg/mL, αIL1α), or IL1β (1 ng/mL, αIL1β) antibody. Results displayed as mean ± SEM, n=4 (IgG/αIL1α), n=3 (αIL1β), one-way ANOVA Tukey test. Source data are provided as a Source Data file.

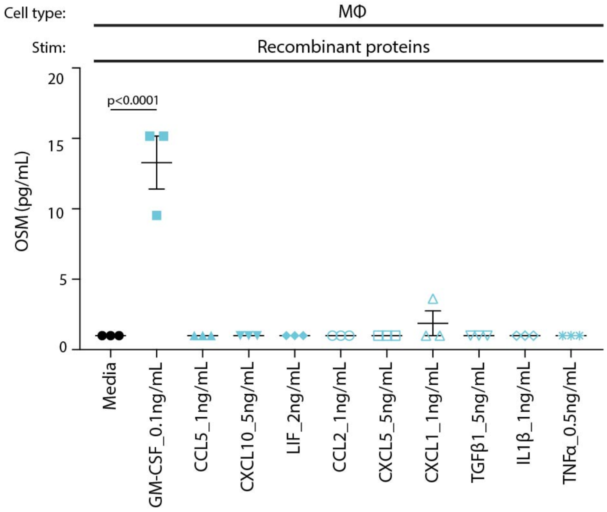

Cell type:
MΦ
Stim:
Recombinant proteins
OSM (pg/mL)
20
15
10
5
0
p<0.0001
Media
GM-CSF_0.1ng/mL
CCL5_1ng/mL
CXCL10_5ng/mL
LIF_2ng/mL
CCL2_1ng/mL
CXCL5_5ng/mL
CXCL1_1ng/mL
TGFβ1_5ng/mL
IL1β_1ng/mL
TNFα_0.5ng/mL

**Supplementary Figure 4. Tumour cell-secreted GM-CSF increases macrophage secretion of OSM**

Quantification of OSM by ELISA in conditioned medium of bone marrow-derived MØs stimulated with recombinant proteins as indicated. Results displayed as mean ± SEM, n=3, one-way ANOVA Tukey test. Source data are provided as a Source Data file.

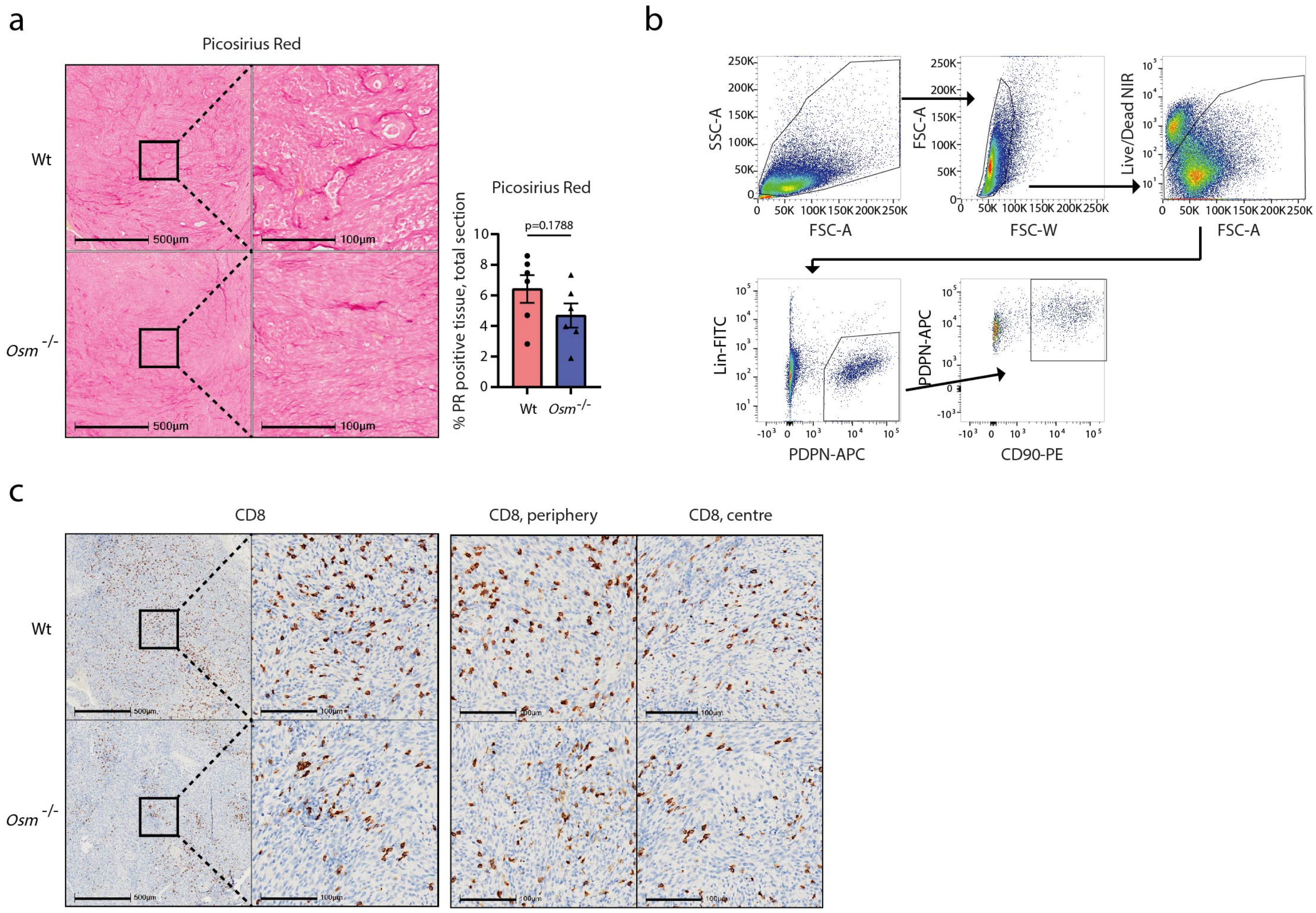
a
Picosirius Red
Wt
Osm -/-
500μm
100μm
Picosirius Red
p=0.1788
% PR positive tissue, total section
10
8
6
4
2
0
Wt
Osm -/-
b
SSC-A
FSC-A
FSC-A
FSC-W
Live/Dead NIR
FSC-A
250K
200K
150K
100K
50K
0
50K
100K
150K
200K
250K
Lin-FITC
PDPN-APC
PDPN-APC
CD90-PE
c
CD8
CD8, periphery
CD8, centre
Wt
Osm -/-
500μm
100μm

**Supplementary Figure 5. OSM regulates CD8 T cell infiltration**

**a) LEFT**: Representative Picosirious Red staining of wildtype and *Osm*$^{-/-}$ tumour sections. **RIGHT**: Quantification of Picosirious Red$^{pos}$ staining of tumour sections of wildtype (n=6) and *Osm*$^{-/-}$ (n=6) animals. Results displayed as mean ± SEM, two-tailed student *t* test.

**b)** Gating strategy for isolating CAFs from tumours **c) LEFT**: Representative CD8 staining of wildtype (n= 6) and *Osm*$^{-/-}$ (n= 6) tumour sections. **RIGHT**: Representative CD8 staining at the periphery and centre of wildtype (n= 6) and *Osm*$^{-/-}$ (n= 6) tumour sections. Source data are provided as a Source Data file.

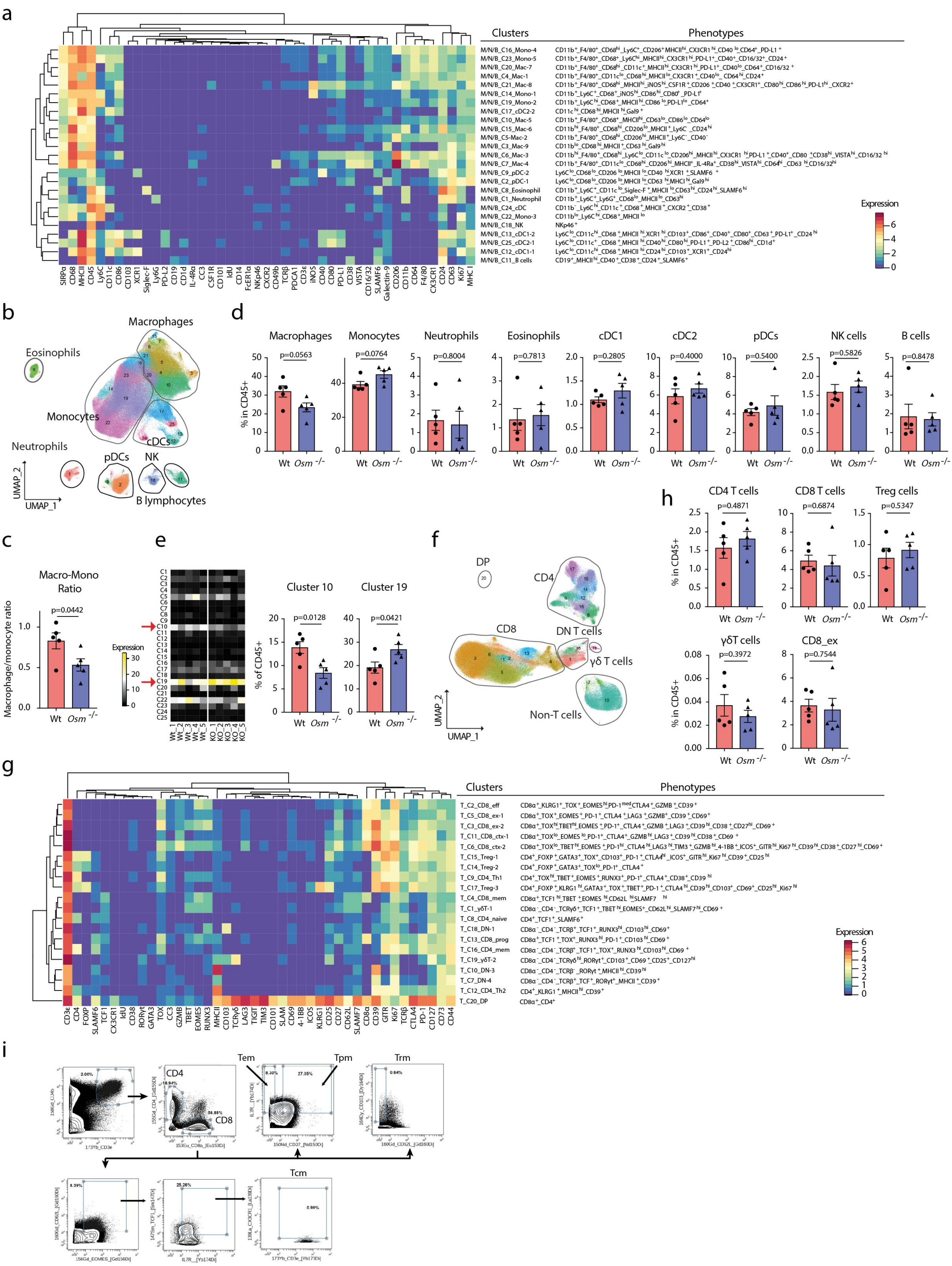
a
Clusters
Phenotypes
M/N/B_C16_Mono-4
M/N/B_C23_Mono-5
M/N/B_C20_Mac-7
M/N/B_C4_Mac-1
M/N/B_C21_Mac-8
M/N/B_C14_Mono-1
M/N/B_C19_Mono-2
M/N/B_C17_cDC2-2
M/N/B_C10_Mac-5
M/N/B_C15_Mac-6
M/N/B_C5-Mac-2
M/N/B_C3_Mac-9
M/N/B_C6_Mac-3
M/N/B_C7_Mac-4
M/N/B_C9_pDC-2
M/N/B_C2_pDC-1
M/N/B_C8_Eosinophil
M/N/B_C1_Neutrophil
M/N/B_C24_cDC
M/N/B_C22_Mono-3
M/N/B_C18_NK
M/N/B_C13_cDC1-2
M/N/B_C25_cDC2-1
M/N/B_C12_cDC1-1
M/N/B_C11_B cells
Expression
b
Macrophages
Eosinophils
Monocytes
cDCs
Neutrophils
pDCs
NK
B lymphocytes
UMAP_1
UMAP_2
d
Macrophages
Monocytes
Neutrophils
Eosinophils
cDC1
cDC2
pDCs
NK cells
B cells
p=0.0563
p=0.0764
p=0.8004
p=0.7813
p=0.2805
p=0.4000
p=0.5400
p=0.5826
p=0.8478
% in CD45+
Wt Osm -/-
c
Macro-Mono Ratio
p=0.0442
Macrophage/monocyte ratio
e
Expression
Cluster 10
Cluster 19
p=0.0128
p=0.0421
% of CD45+
f
DP
CD4
CD8
DN T cells
γδ T cells
Non-T cells
h
CD4 T cells
CD8 T cells
Treg cells
p=0.4871
p=0.6874
p=0.5347
γδT cells
CD8_ex
p=0.3972
p=0.7544
% in CD45+
g
Clusters
Phenotypes
T_C2_CD8_eff
T_C5_CD8_ex-1
T_C3_CD8_ex-2
T_C11_CD8_ctx-1
T_C6_CD8_ctx-2
T_C15_Treg-1
T_C14_Treg-2
T_C9_CD4_Th1
T_C17_Treg-3
T_C4_CD8_mem
T_C1_γδT-1
T_C8_CD4_naive
T_C18_DN-1
T_C13_CD8_prog
T_C16_CD4_mem
T_C19_γδT-2
T_C10_DN-3
T_C7_DN-4
T_C12_CD4_Th2
T_C20_DP
Expression
i
Tem
Tpm
Trm
CD4
CD8
Tcm

**Supplementary Figure 6. OSM supports the formation of an immunosuppressive microenvironment**

**a)** Annotation of FlowSOM clusters to unique myeloid/NK/B cell (M/N/B) populations. Heatmap shows M/N/B marker expression in wildtype (n=5) and *Osm*$^{-/-}$ (n=5) tumours. **b)** Self-organising map clustering (FlowSOM) of 250,000 M/N/B cells from 10 tumours (wildtype (n=5) vs *Osm*$^{-/-}$ (n=5) tumours) displayed in a UMAP plot. **c)** Macrophage-monocyte ratio displayed for wildtype (n=5) and *Osm*$^{-/-}$ (n=5) tumours. Results displayed as mean ± SEM, two-tailed student *t* test. **d)** Frequency of indicated M/N/B cell types in wildtype (n=5) and *Osm*$^{-/-}$ (n=5) tumours. Results displayed as mean ± SEM, two-tailed student *t* test. **e)** Quantification of FlowSOM cluster abundances in wildtype (n=5) and *Osm*$^{-/-}$ (n=5) tumours. Results displayed as mean ± SEM, two-tailed student *t* test. **f)** Self-organising map clustering (FlowSOM) of 182,800 T cells from 10 tumours (wildtype (n=5) vs *Osm*$^{-/-}$ (n=5) tumours) displayed in a UMAP plot. **g)** Annotation of FlowSOM clusters to unique T cell populations. Heatmap shows FlowSOM cluster expression of T cell markers in wildtype (n=5) and *Osm*$^{-/-}$ (n=5) tumours. **h)** Frequency of T cell types in wildtype (n=5) and *Osm*$^{-/-}$ (n=5) tumours. Results displayed as mean ± SEM, two-tailed student *t* test. **i)** Gating strategy for characterisation of memory T cell populations in wildtype (n=5) and *Osm*$^{-/-}$ (n=5) tumours. Source data are provided as a Source Data file.

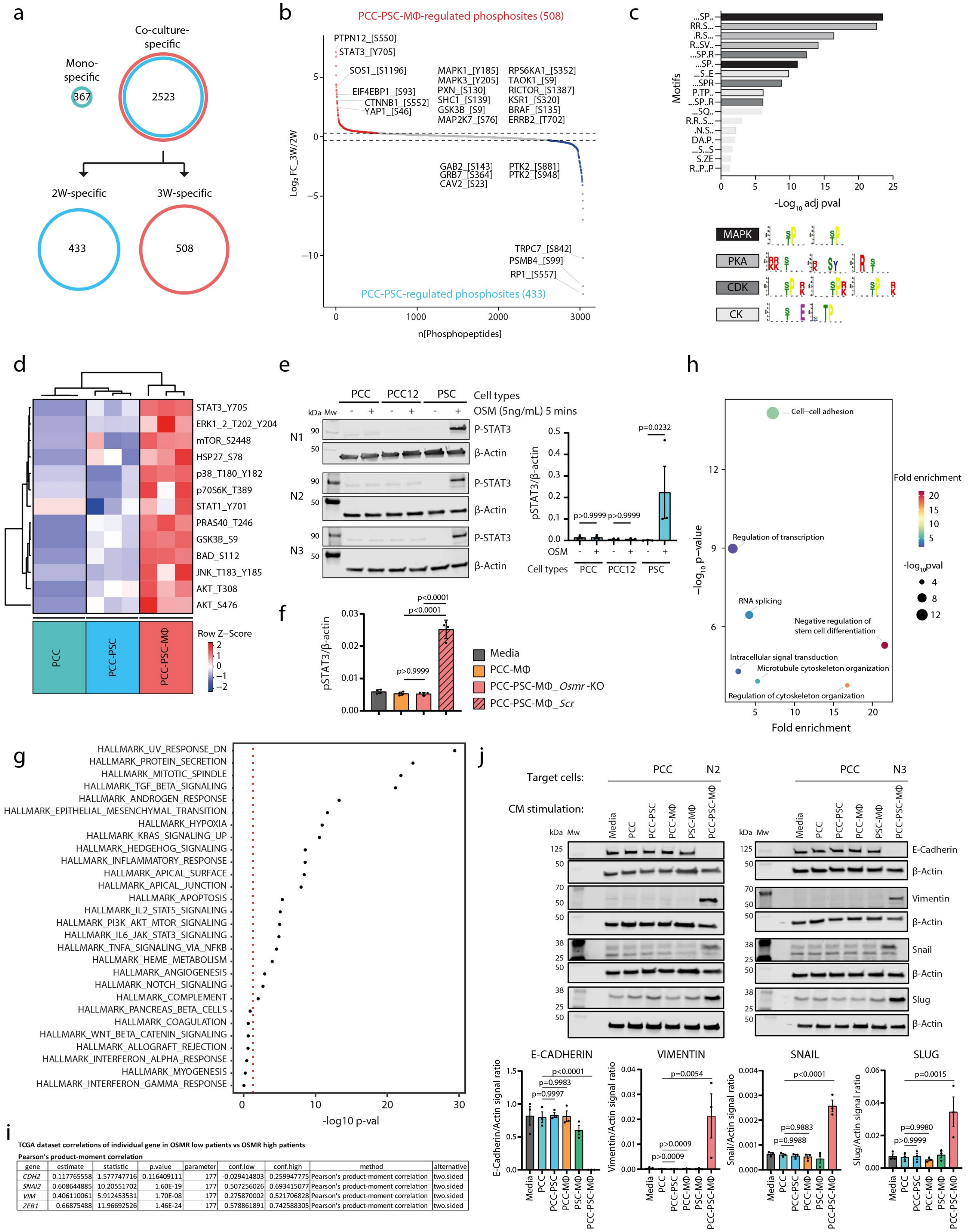


**TCGA dataset correlations of individual gene in OSMR low patients vs OSMR high patients**

**Pearson's product-moment correlation**

| gene | estimate | statistic | p.value | parameter | conf.low | conf.high | method | alternative |
|---|---|---|---|---|---|---|---|---|
| *CDH2* | 0.117765558 | 1.577747716 | 0.116409111 | 177 | -0.029414803 | 0.259947775 | Pearson's product-moment correlation | two.sided |
| *SNAI2* | 0.608644885 | 10.20551702 | 1.60E-19 | 177 | 0.507256026 | 0.693415077 | Pearson's product-moment correlation | two.sided |
| *VIM* | 0.406110061 | 5.912453531 | 1.70E-08 | 177 | 0.275870002 | 0.521706828 | Pearson's product-moment correlation | two.sided |
| *ZEB1* | 0.66875488 | 11.96692526 | 1.46E-24 | 177 | 0.578861891 | 0.742588305 | Pearson's product-moment correlation | two.sided |

**Supplementary Figure 7. PCC-PSC-MØ interactions reshape tumour cell signalling**

SILAC-based phosphoproteomics analysis of stroma-regulated tumour cell signalling. **a)** Number of regulated PCC phosphopeptides under conditions as indicated using following criteria: < 1 % FDR; > 75 % phosphorylation site localisation probability, detected in > 2 biological replicates, > $\log_2$FC 0.3 or < $\log_2$FC -0.3. **b)** Waterfall plot displaying PCC-PSC- or PCC-PSC-MØ-regulated PCC phosphosites. Results displayed as mean (n=5). **c)** Motif enrichment analysis of upregulated (3W/2W, log2FC > 0.3) PCC-PSC-MØ-directed tumour cell phosphopeptides. **d)** Targeted phosphorylation analysis showing PCC-, PCC-PSC- and PCC-PSC-MØ-regulated tumour cell signalling, as measured by phosphoarray. PCC-PSC-MØ-induced activation of STAT3, ERK1/2, AKT, p38, JNK is beyond that of PCC and PCC-PSC signals (n=3 per culture condition). **e)** Induced phospho-STAT3 levels in PCC, PCC12 and PSCs following recombinant murine OSM stimulation (5 ng/mL). **LEFT:** western blots, n=3. **RIGHT:** Quantification, results displayed as mean ± SEM of 3 biological replicates, one-way ANOVA Tukey test. **f)** Quantification of phospho-STAT3 protein levels in PCC following stimulation with condition medium generated from 2W or 3W co-cultures containing *Osmr*-KO or *Scr*-PSCs. Results displayed as mean ± SEM of 4 biological replicates, one-way ANOVA Tukey test. **g)** Tumour purity independence analysis of enriched Hallmarks gene sets in *OSMR*$^{pos}$ PDA patients in comparison to *OSMR*$^{neg}$ patients from TCGA PanCancer PAAD dataset (n=179). Enriched Hallmark gene sets on the right side of the red dotted line are significantly correlated with *OSMR* expression in PDA patients as well as being independent of tumour purity. **h)** Biological process enrichment analysis (DAVID) of PCC-PSC-MØ-regulated tumour cell signalling (3W/2W, log2FC > 0.3). **i)** Correlations of individual EMT gene markers between *OSMR* low and *OSMR* high patients, using TCGA PAAD dataset. **j)** EMT marker expression in PCCs treated with conditioned medium from 1W, 2W or 3W co-cultures, E-cadherin, Vimentin, SNAIL and SLUG. **UPPER:** western blots from biological replicates 2 and 3. **LOWER:** quantification of the detected protein levels. Results displayed as mean ± SEM of 3 biological replicates, one-way ANOVA Tukey test. Source data are provided as a Source Data file.

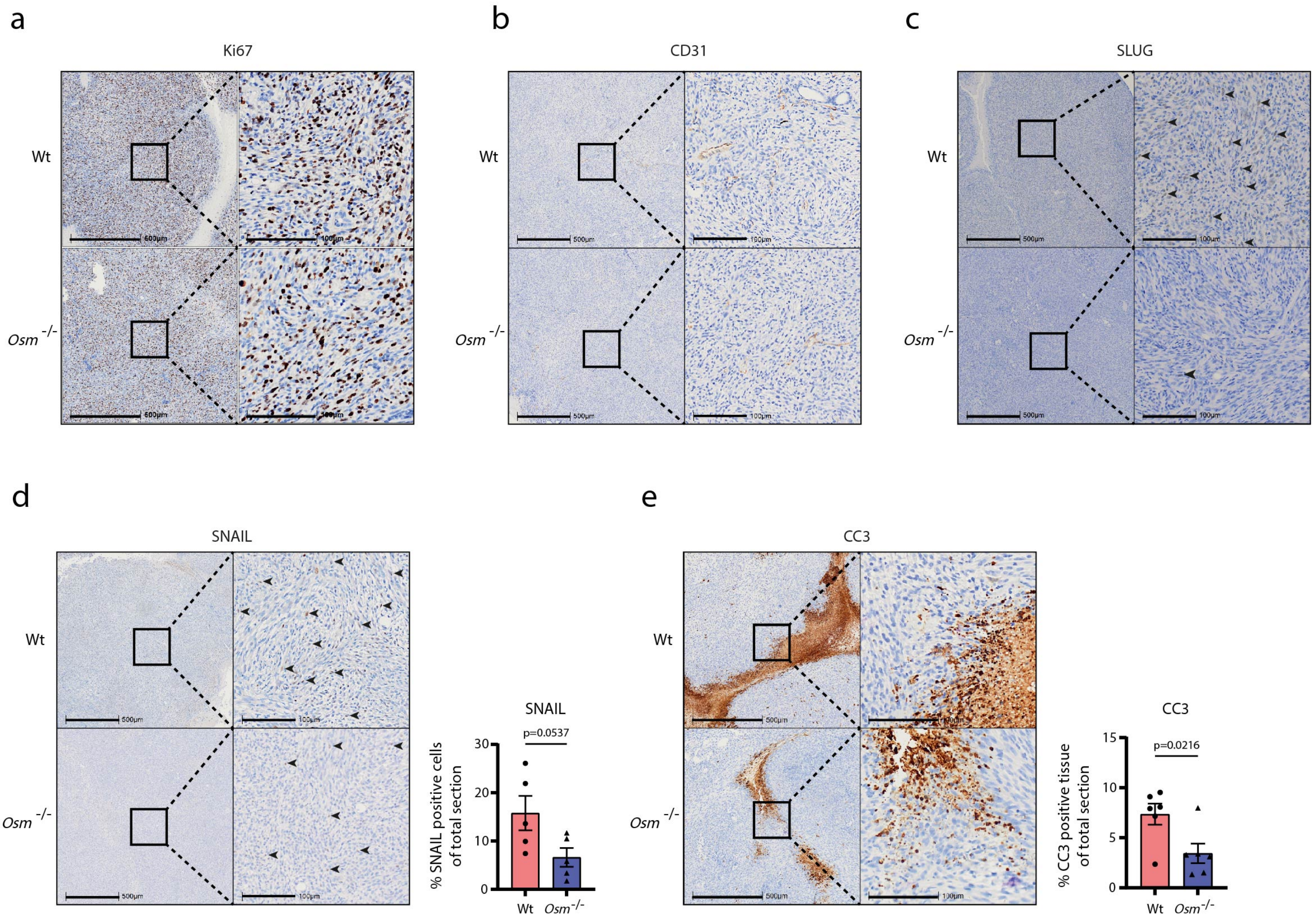

a
Ki67
Wt
Osm -/-
b
CD31
Wt
Osm -/-
c
SLUG
Wt
Osm -/-
d
SNAIL
Wt
Osm -/-
SNAIL
p=0.0537
% SNAIL positive cells of total section
30
20
10
0
Wt
Osm -/-
e
CC3
Wt
Osm -/-
CC3
p=0.0216
% CC3 positive tissue of total section
15
10
5
0
Wt
Osm -/-

**Supplementary Figure 8. OSM drives tumour growth and metastasis in vivo**

**a)** Representative Ki67 immunohistochemical staining of wildtype (n= 6) and *Osm*$^{-/-}$ (n= 6) sections. **b)** Representative CD31 immunohistochemical staining of wildtype (n= 6) and *Osm*$^{-/-}$ (n= 6) tumour sections. **c)** Representative SLUG immunohistochemical staining of wildtype (n= 6) and *Osm*$^{-/-}$ (n= 6) tumour sections. Arrow heads indicate a selection of SLUG$^{pos}$ cells. **d) LEFT:** Representative SNAIL immunohistochemical staining of wildtype and *Osm*$^{-/-}$ tumour sections. Arrow heads indicate a selection of SNAIL$^{pos}$ cells. **RIGHT:** Quantification of SNAIL$^{pos}$ staining of tumour sections of wildtype (n=5) and *Osm*$^{-/-}$ (n=5) animals. Results displayed as mean ± SEM, two-tailed student *t* test. **e) LEFT:** Representative cleaved caspase 3 (CC3) immunohistochemical staining of wildtype and *Osm*$^{-/-}$ tumour sections. **RIGHT:** Quantification of CC3$^{pos}$ tissue area of tumour sections of wildtype (n=6) and *Osm*$^{-/-}$ (n=6) animals. Results displayed as mean ± SEM, two-tailed student *t* test. Source data are provided as a Source Data file.

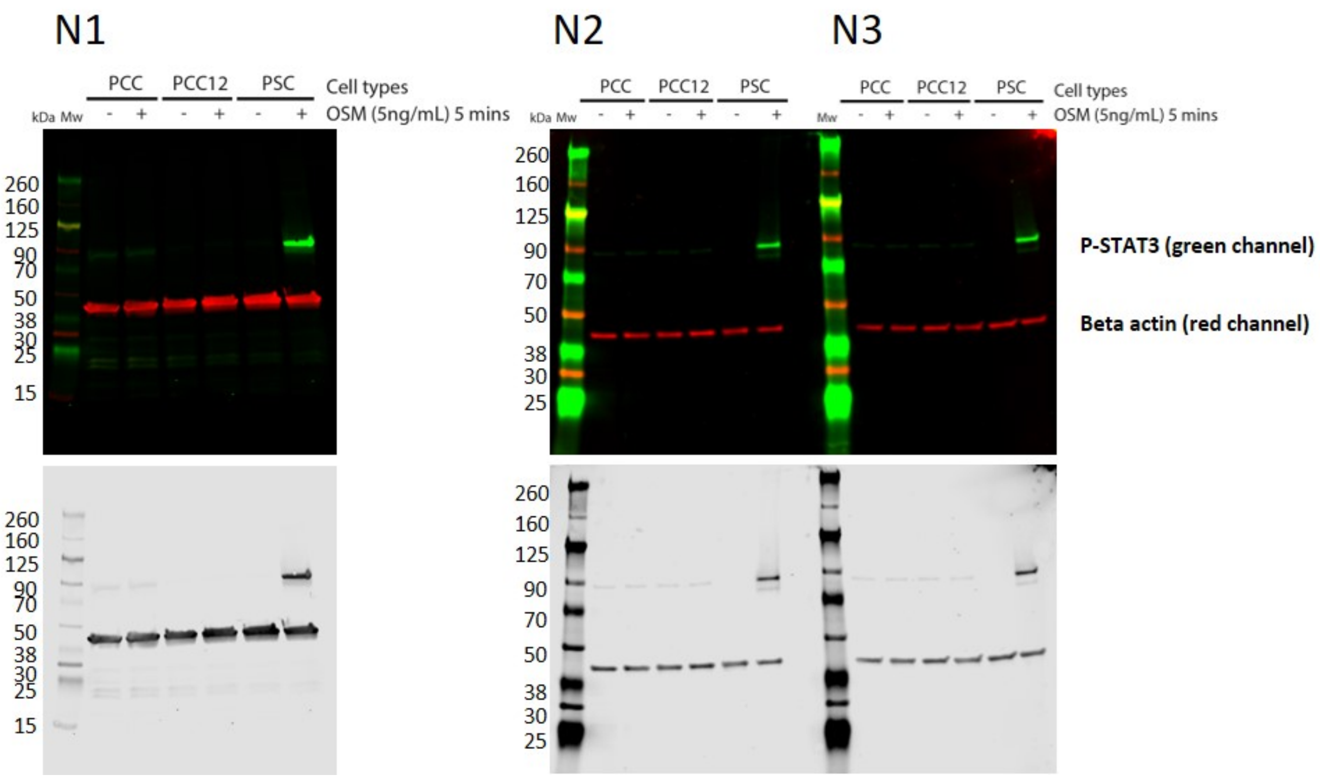
N1
N2
N3
PCC
PCC12
PSC
Cell types
OSM (5ng/mL) 5 mins
kDa Mw
260
160
125
90
70
50
38
30
25
15
P-STAT3 (green channel)
Beta actin (red channel)

**Supplementary Figure 9. Uncropped western blots referring to Supplementary Figure 7e.**

Induced phospho-STAT3 levels in PCC, PCC12 and PSCs following recombinant murine OSM stimulation (5 ng/mL). Loading control β-actin. Three independent biological replicates.

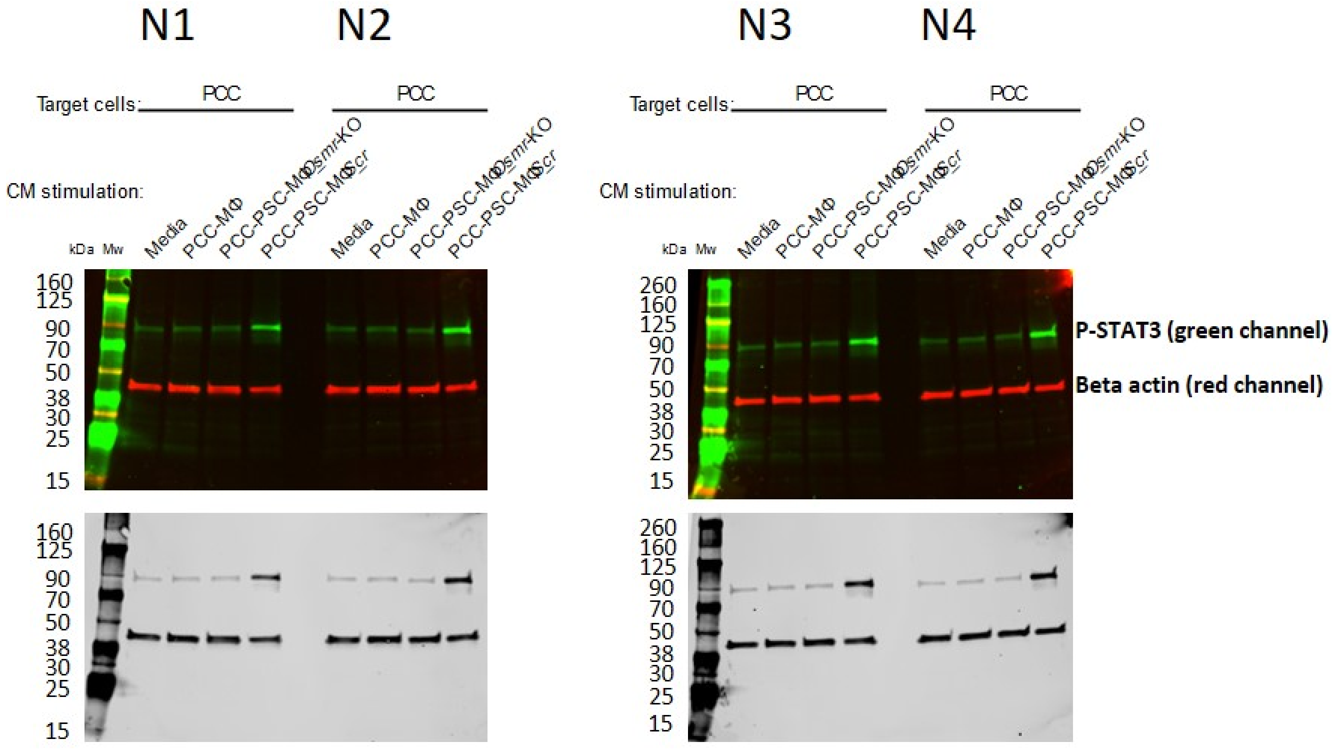
N1
N2
N3
N4
Target cells:
PCC
CM stimulation:
Media
PCC-MΦ
PCC-PSC-MΦ_Osmr-KO
PCC-PSC-MΦ_Scr
kDa
Mw
260
160
125
90
70
50
38
30
25
15
P-STAT3 (green channel)
Beta actin (red channel)

**Supplementary Figure 10. Uncropped western blots referring to Supplementary Figure 7f.**

Phospho-STAT3 levels in PCC following stimulation with condition medium generated from 2W or 3W co-cultures containing *Osmr*-KO or *Scr*-PSCs. Loading control β-actin. Four independent biological replicates.

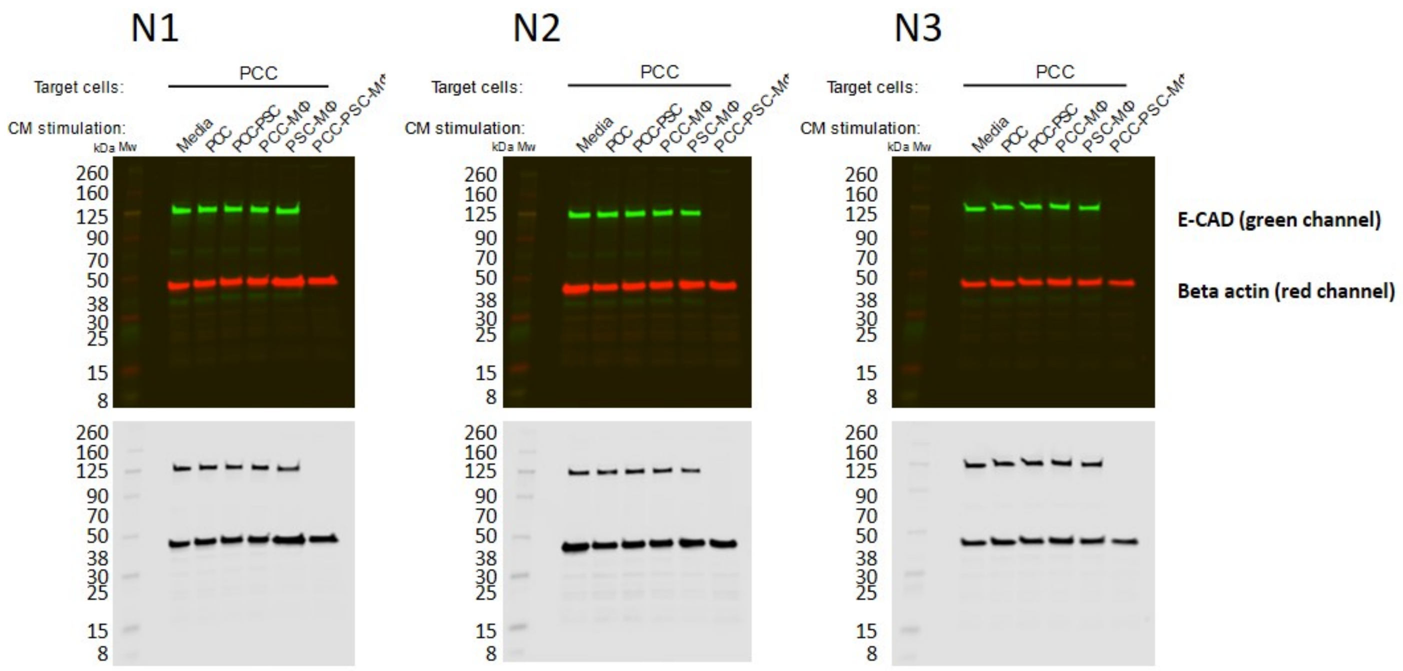
N1
N2
N3
Target cells:
PCC
CM stimulation:
Media
PCC
PCC-PSC
PCC-MΦ
PSC-MΦ
kDa Mw
260
160
125
90
70
50
38
30
25
15
8
E-CAD (green channel)
Beta actin (red channel)

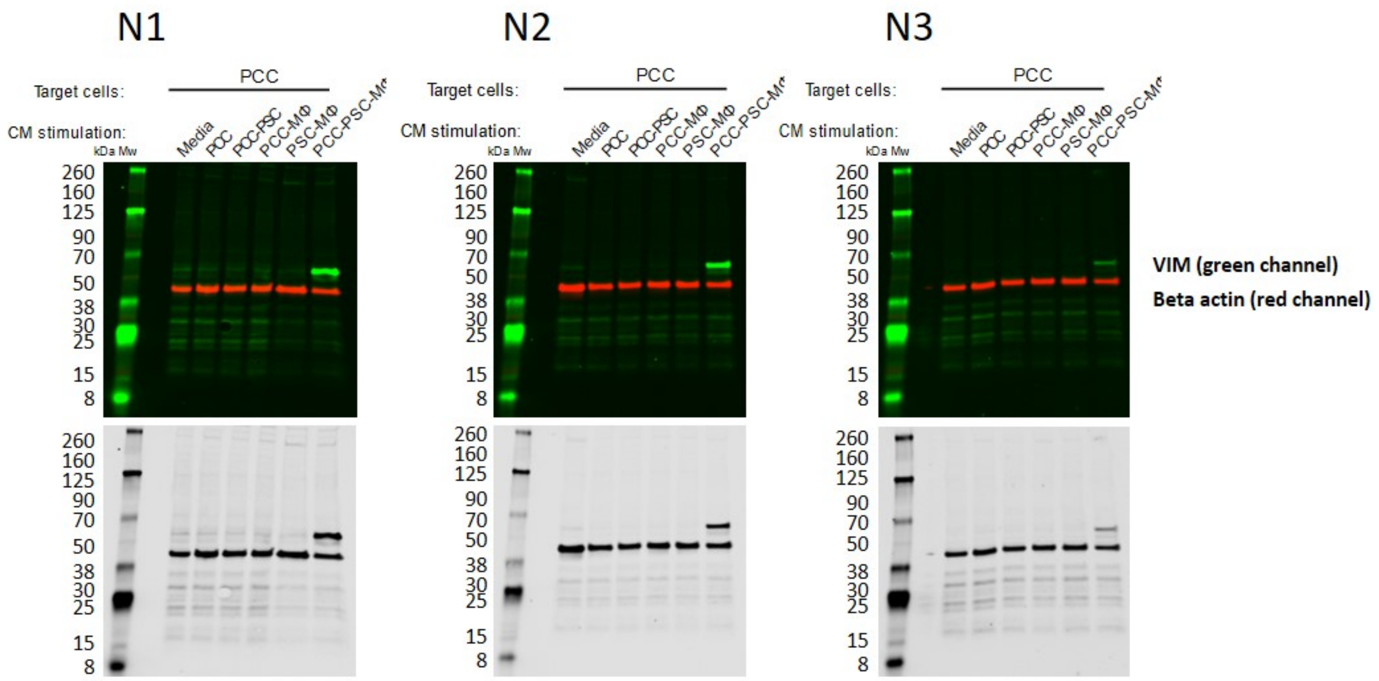
N1
N2
N3
Target cells:
PCC
CM stimulation:
Media
PCC
PCC-PSC
PCC-MΦ
PSC-MΦ
kDa Mw
260
160
125
90
70
50
38
30
25
15
8
VIM (green channel)
Beta actin (red channel)

**Supplementary Figure 11. Uncropped western blots referring to Supplementary Figure 7j.**

EMT marker expression in PCCs treated with conditioned medium from 1W, 2W or 3W co-cultures. Western blots for E-cadherin (E-CAD) and Vimentin (VIM) with loading control β-actin. Three independent biological replicates.

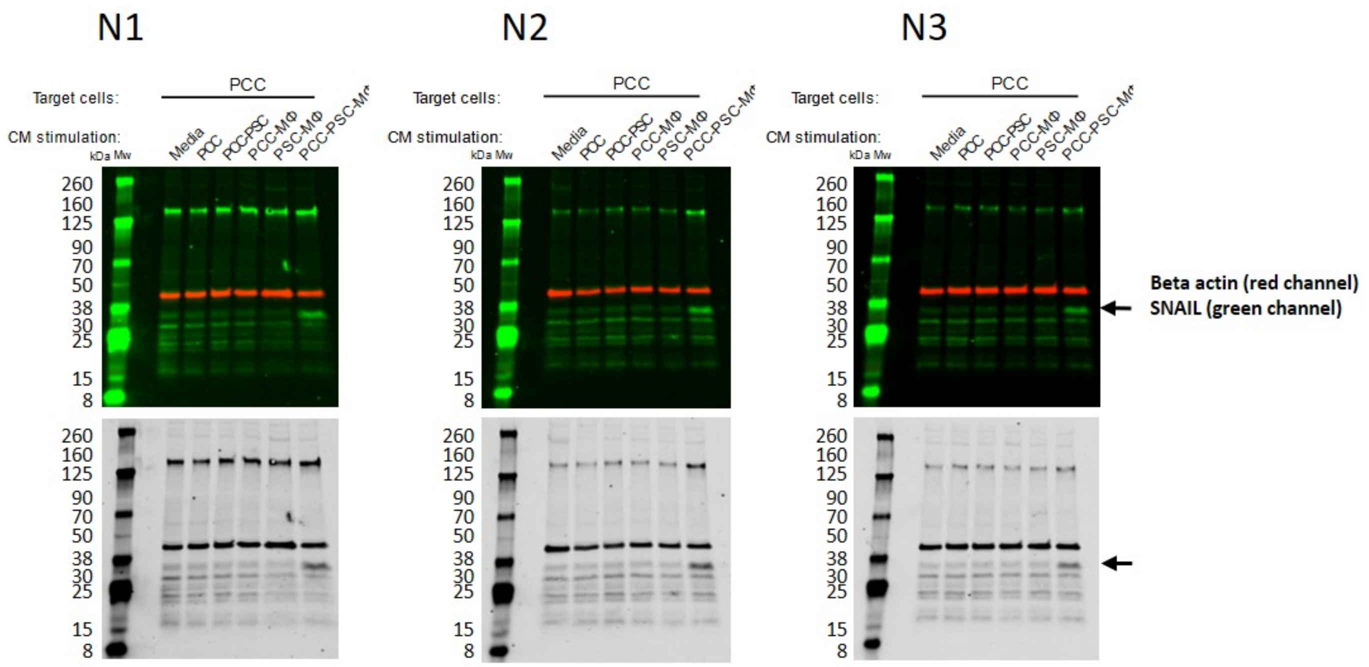
N1
N2
N3
Target cells:
PCC
CM stimulation:
kDa Mw
Media
PCC
PCC-PSC
PCC-MΦ
PSC-MΦ
PCC-PSC-M
260
160
125
90
70
50
38
30
25
15
8
Beta actin (red channel)
SNAIL (green channel)

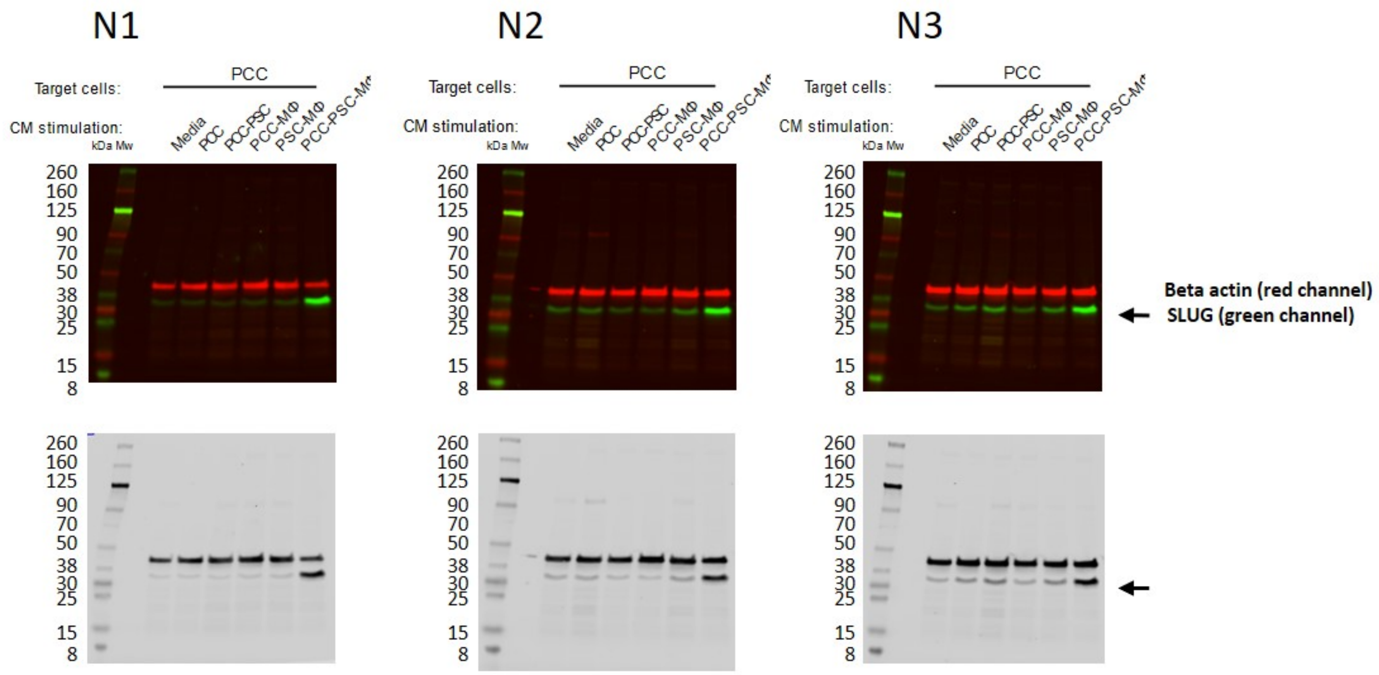
N1
N2
N3
Target cells:
PCC
CM stimulation:
kDa Mw
Media
PCC
PCC-PSC
PCC-MΦ
PSC-MΦ
PCC-PSC-M
260
160
125
90
70
50
38
30
25
15
8
Beta actin (red channel)
SLUG (green channel)

**Supplementary Figure 12. Uncropped western blots referring to Supplementary Figure 7j.**

EMT marker expression in PCCs treated with conditioned medium from 1W, 2W or 3W co-cultures. Western blots for SNAIL and SLUG, with loading control β-actin. Three independent biological replicates.

imPSC.C2 wildtype

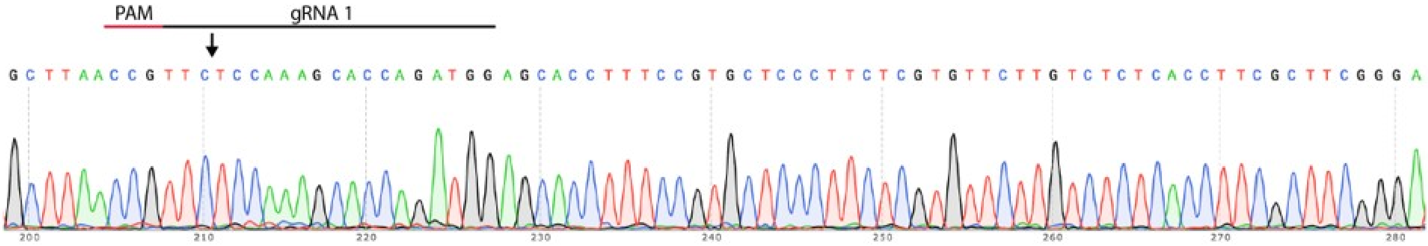


imPSC.C2 OSMR-KO1

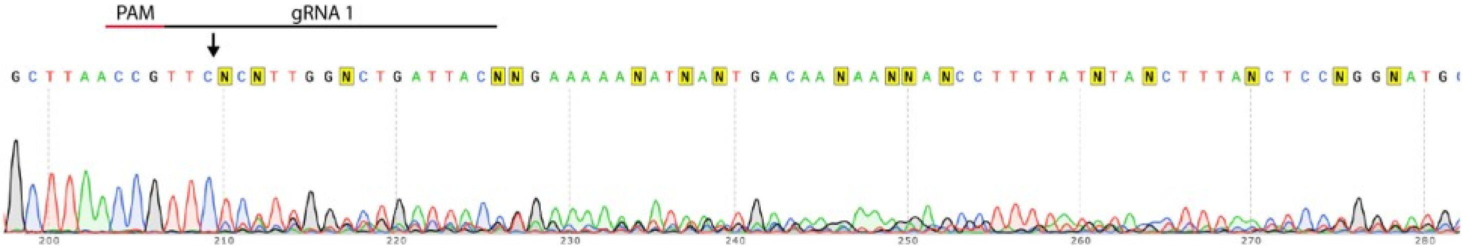


imPSC.C2 OSMR-KO2

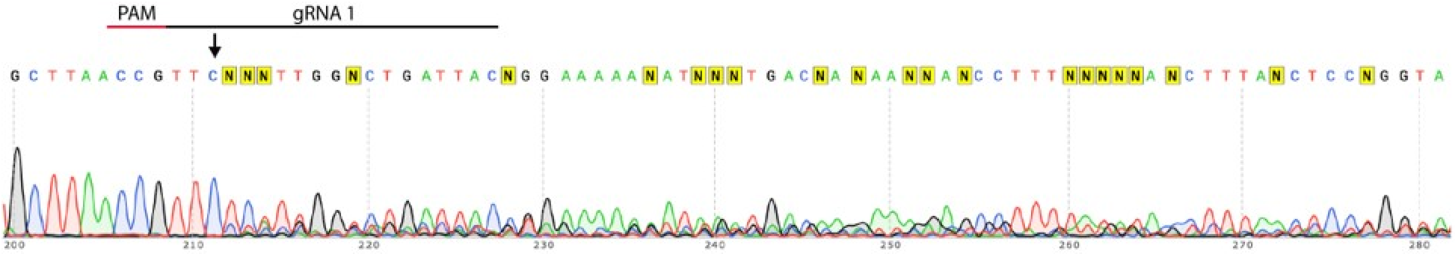


imPSC.C2 OSMR-KO3

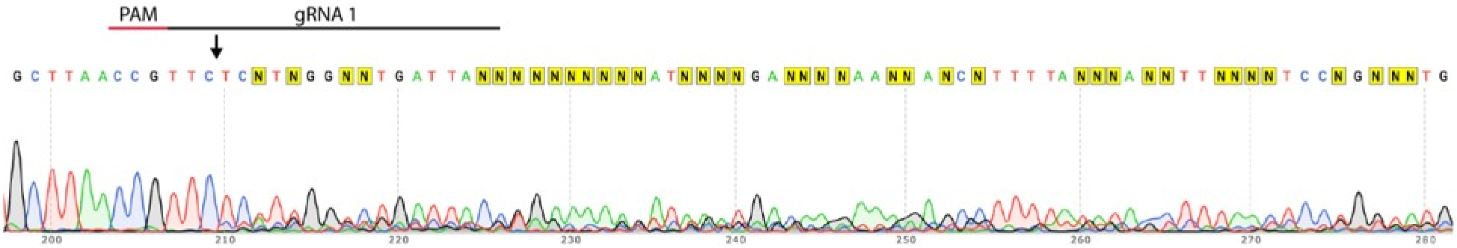

**Supplementary Figure 13. Sanger sequencing validation of *Osmr*-KO PSCs**

Sanger sequencing validation of the CRISPR knockout (KO) of *Osm* in murine PSCs. PAM and guide RNA sites indicated.

## SUPPLEMENTARY TABLES:

## Supplementary Table 1. Mass cytometry fibroblast antibody panel, including extracellular and intracellular targets.

**Extracellular**

| Antibody target | Metal | Clone | Pre-conjugated/custom | Supplier | Product no |
|---|---|---|---|---|---|
| Fc block | - | 2.4G2 | - | BD Biosciences | 558636 |
| CD44 | 114Cd | IM7 | Custom | Biolegend | 103002 |
| MCAM | 141Pr | ME-9F1 | Pre-conjugated | Fluidigm | 3141016B |
| ITGA5 | 142Nd | H10-27(MFR5 | Custom | Biolegend | 103801 |
| CD81 | 143Nd | Eat-2 | Custom | Biolegend | 104902 |
| CD87 | 144Nd | 109801 | Custom | Thermo Fisher | MA5-23853 |
| PE (ITGA1) | 176Yb | PE001 | Pre-conjugated | Fluidigm | 3145006B |
| ITGAV | 146Nd | RMV-7 | Custom | Biolegend | 104102 |
| ITGA2 | 147Sm | Hma2 | Custom | Biolegend | 103501 |
| PDGFRA | 148Nd | APA5 | Pre-conjugated | Fluidigm | 3148018B |
| PDPN | 149Sm | 8.1.1 | Custom | Biolegend | 127402 |
| CD24 | 150Nd | M1/69 | Pre-conjugated | Fluidigm | 3150009B |
| PDGFRB | 151Eu | APB5 | Pre-conjugated | Fluidigm | 3151017B |
| ICAM1 | 153Eu | YN1/1.7.4 | Custom | Biolegend | 116102 |
| CD73 | 156Gd | TY/11.8 | Custom | Biolegend | 127202 |
| ITGB3 | 161Dy | c9.G2 (HMB3 | Custom | Biolegend | 104302 |
| CD34 | 162Dy | MEC14.7 | Custom | Biolegend | 119302 |
| ITGA6 | 164Dy | GoH3 | Custom | Biolegend | 313602 |
| Biotin (CD105) | 165Ho | 1D4-C5 | Pre-conjugated | Fluidigm | 3165012B |
| CD14 | 155Gd | Sa14-2 | Custom | Biolegend | 123302 |
| CD47 | 168Di | In1/CD74 | Custom | Biolegend | 151002 |
| GFP | 170Di | FM264G | Custom | Biolegend | 338002 |
| CD38 | 171Yb | 90 | Pre-conjugated | Fluidigm | 3171007B |
| ITGB1 | 163Dy | HMB1-1 | Custom | Biolegend | 102202 |
| | 166Er | | | | |
| | 169Tm | | | | |
| | 172Yb | | | | |
| | 115In | | | | |
| VCAM1 | 173Yb | 29 (MVCAM.A | Custom | Biolegend | 105702 |
| CD45 | 175Lu | 30-F11 | Pre-conjugated | Fluidigm | 3175010B |
| APC (CD90) | 160Gd | APC003 | Pre-conjugated | Fluidigm | 3176007B |
| MHCII | 209Bi | M5/114.15.2 | Pre-conjugated | Fluidigm | 3209006B |
| CD47 | 168Er | MIAP201 | Custom | Biolegend | 127602 |

**Intracellular**

| Antibody target | Metal | Clone | Pre-conjugated/custom | Supplier | Identifier |
|---|---|---|---|---|---|
| Fc block | - | 2.4G2 | - | BD Biosciences | 558636 |
| PDPN | 149Sm | 8.1.1 | Custom | Biolegend | 127402 |
| Pan-cytokeratin | 152Sm | C-11 | Custom | Biolegend | 628602 |
| VIM | 154Sm | D21H3 | Pre-conjugated | Fluidigm | 3154014A |
| DES | 158Gd | Y66 | Custom | Abcam | ab271829 |
| aSMA | 159Tb | 1A4 | Custom | Abcam | ab240654 |
| Collagen-4 | 174Yb | n/a (pAb) | Custom | Abcam | ab6586 |

## Supplementary Table 2. Mass cytometry Myeloid/NK/B cell antibody panel, including extracellular and intracellular targets.

**Extracellular**

| Metal | Target | Vol (uL) per 3x10^6 cells | Clone | Manufacturer | Product no |
|---|---|---|---|---|---|
| 151Eu | CD64 | 1 | X54-5/7.1 | Fluidigm | 3151012B |
| 145Nd | CD16/CD32 | 1 | 93 | Biolegend | 101302 |
| 089Y | CD11b | 1 | M1/70 | Biolegend | 101202 |
| 113In | PDCA1 | 1 | 927 | Biolegend | 127002 |
| e115In | CD68 | 1 | FA-11 | Biolegend | 137002 |
| 139La | CD14 | 1 | Sa14-2 | Biolegend | 123302 |
| 141Pr | Ly6G | 1 | 1A8 | Fluidigm | 3141008B |
| 143Nd | Siglec-F | 1 | E50-2440 | BD Biosciences | 552125 |
| 144Nd | PD-L1 | 1 | 10F.9G2 | Biolegend | 124302 |
| 146Nd | F4/80 | 1 | BM8 | Fluidigm | 3146008B |
| 147Sm | MHCI | 1 | 28-14-8 | Biolegend | 114502 |
| 148Nd | CD3e | 1 | 17A2 | BD Biosciences | 555273 |
| 149Sm | CD19 | 1 | 6D5 | Fluidigm | 3149002B |
| 150Nd | CD1d | 1 | 1B1 | Biolegend | 123502 |
| 154Sm | CD11c | | N418 | Biolegend | 117302 |
| 155Gd | CD63 | 1 | NVG-2 | Biolegend | 143902 |
| 156Gd | XCR1 | 1 | ZET | Biolegend | 148202 |
| 157Gd | TCRb | 1 | H57-597 | Biolegend | 109202 |
| 158Gd | CD45 | 1 | 30-F11 | Biolegend | 103102 |
| 159Tb | CX3CR1 | 1 | SA011F11 | Biolegend | 149002 |
| 160Gd | CXCR2 | 1 | SA044G4 | Biolegend | 149302 |
| 162Dy | CSF1R | 1 | AFS98 | Biolegend | 135502 |
| 163Dy | CD40 | 1 | HM40-3 | Biolegend | 102902 |
| 164Dy | CD103 | 1 | 2.00E+07 | Biolegend | 121402 |
| 165Ho | PD-L2 | 1 | TY25 | Biolegend | 107202 |
| 166Er | VISTA | 1 | MIH63 | Biolegend | 150202 |
| 167Er | SIRPa | 1 | P84 | Biolegend | 144002 |
| 168Er | IL-4Ra | 1 | I015F8 | Biolegend | 144802 |
| 169Tm | CD206 | 1 | C086C2 | Fluidigm | 3169021B |
| 170Er | CD49b | 1 | HMa2 | Fluidigm | 3170008B |
| 171Yb | CD80 | 1 | 16-10A1 | Fluidigm | 3171008B |
| 172Yb | CD86 | 1 | GL1 | Fluidigm | 3172016B |
| 173Yb | CD101 | 1 | Moushi101 | Biolegend | Custom order |
| 174Yb | NKp46 | 1 | 29A1.4 | Biolegend | 137602 |
| 175Lu | CD38 | 1 | 90 | Fluidigm | 3175014B |
| 176Yb | Ly6C | 1 | HK1.4 | Biolegend | 128002 |
| 194Pt | CD24 | 1 | M1/69 | Biolegend | 101802 |
| 195Pt | SLAM F6 | 1 | TC15-12F12.2 | Biolegend | 115902 |
| 209Bi | MHCII | 1 | M5/114.15.2 | Fluidigm | 3209006B |

**Intracellular**

| Metal | Target | Vol (uL) per 3x10^6 cells | Clone | Manufacturer | Product no |
|---|---|---|---|---|---|
| 142Nd | CC3 | 1 | D3E9 | Cell Signaling Technology | 9579 |
| 152Sm | Ki67 | 1 | So1A15 | Thermo Fisher | 14-5698-82 |
| 153Eu | Galectin-9 | 1 | 9M1-3 | Thermo Fisher | 16-9116-85 |
| 155Gd | CD63 | 1 | NVG-2 | Biolegend | 143902 |
| 161Dy | iNOS | 1 | CXNFT | Fluidigm | 3161011B |

## Supplementary Table 3. Mass cytometry T cell antibody panel, including extracellular and intracellular targets.

**Extracellular**

| Metal | Target | Amount per 3x10^6 cells | Clone | Manufacturer | Product no |
|---|---|---|---|---|---|
| 089Y | Ly6C | 1 | HK1.4 | Biolegend | 128002 |
| 115In | TCRb | 1 | H57-597 | Biolegend | 109202 |
| 139La | CX3CR1 | 1 | SA011F11 | Biolegend | 149002 |
| 143Nd | GITR | 1 | DTA1 | Fluidigm | 3143019B |
| 144Nd | CD44 | 1 | IM7 | Biolegend | 103002 |
| 145Nd | CD69 | 1 | H1.2F3 | Fluidigm | 3145005B |
| 146Nd | TIGIT | 1 | 1G9 | Biolegend | 142102 |
| 148Nd | 4-1BB | 1 | 17B5 | Biolegend | 106107 |
| 149Sm | CD101 | 1 | Moushi101 | Biolegend | Custom order |
| 150Nd | CD27 | 1 | LG.3A10 | Fluidigm | 3150017B |
| 151Eu | LAG3 | 1 | C9B7W | Biolegend | 125202 |
| 153Eu | CD8a | 1 | 53-6.7 | Fluidigm | 3153012B |
| 154Sm | CTLA4 | 1 | UC10-4B9 | Fluidigm | 3154008B |
| 155Gd | CD4 | 1 | RM4-5 | Biolegend | 100506 |
| 157Gd | SLAM F7 | 1 | TC15-12F12.2 | Biolegend | 115902 |
| 158Gd | CD45 | 1 | 30-F11 | Fluidigm | 3175010B |
| 159Tb | PD-1 | 1 | 29F.1A12 | Fluidigm | 3159024B |
| 160Gd | CD62L | 1 | MEL-14 | Fluidigm | 3160008B |
| 162Dy | TIM3 | 1 | RMT3-23 | Fluidigm | 3162029B |
| 163Dy | CD49b | 1 | HMa2 | Biolegend | 103501 |
| 164Dy | CD103 | 1 | 20000000 | Biolegend | 121402 |
| 167Er | KLRG1 | 1 | 2F1 | BD Biosciences | 562190 |
| 168Er | ICOS | 1 | C398.4A | Fluidigm | 3168024B |
| 169Tm | CD39 | 1 | 24DMS1 | Thermo Fisher | 14-0391-82 |
| 171Yb | SLAM | 1 | TC15-12F12.2 | Biolegend | 115902 |
| 172Yb | CD25 | 1 | PC61 | Biolegend | 102002 |
| 173Yb | CD3e | 1 | 17A2 | BD Biosciences | 555273 |
| 174Yb | CD127 | 1 | A7R34 | Fluidigm | 3174013B |
| 175Lu | CD38 | 1 | 90 | Fluidigm | 3171007B |
| 176Yb | TCRgd | 1 | UC7-13D5 | Biolegend | 107502 |
| 194Pt | CD73 | 1 | TY/11.8 | Biolegend | 127202 |
| 195Pt | SLAM F6 | 1 | TC15-12F12.2 | Biolegend | 115902 |
| 209Bi | MHCII | 1 | M5/114.15.2 | Fluidigm | 3209006B |

**Intracellular**

| Metal | Target | Amount per 3x10^6 cells | Clone | Manufacturer | Product no |
|---|---|---|---|---|---|
| 113In | GATA3 | 1 | L50-823 | BD Biosciences | 558686 |
| 141Pr | GZMB | 1 | GB11 | Thermo Fisher | MA1-80734 |
| 142Nd | CC3 | 1 | D3E9 | Cell Signaling Technology | 9579 |
| 147Sm | TCF1 | 1 | C63D9 | Cell Signaling Technology | 2203 |
| 152Sm | Ki67 | 1 | So1A15 | Thermo Fisher | 14-5698-82 |
| 156Gd | EOMES | 1 | Dan11mag | Thermo Fisher | 14-4875-82 |
| 161Dy | TBET | 1 | 4B10 | Fluidigm | 3160010B |
| 165Ho | FOXP3 | 1 | FJK-16s | Fluidigm | 3165024A |
| 166Er | TOX | 1 | REA473 | Miltenyi Biotech | 130-095-212 |
| 170Er | RUNX3 | 1 | R3-5G4 | Biolegend | 697902 |
| 173Yb | CD3e | 1 | 17A2 | BD Biosciences | 555273 |
| 196Pt | RORgT | 1 | Q31-378 | BD Biosciences | 562663 |

## Supplementary Table 4. Guide RNA sequences for the CRISPR knockout of *Osm.*

| gRNA target gene | Sequence | Manufacturer |
|---|---|---|
| *Scrambled non-targeting_1* | gcacuaccagagcuaacuca | Synthego |
| *Scrambled non-targeting_2* | guacgucgguauaacuccuc | Synthego |
| *Osmr_1* | ACCACAGAGAAAGCCA | Synthego |
| *Osmr_2* | GTCTCTCACCTTCGCTT | Synthego |
| *Osmr_3* | TCTGGTGCTTTGGAGAA | Synthego |

## Supplementary Table 5. Antibody details for immunohistochemistry, immunoblotting and immunofluorescence staining, and the *in situ* hybridisation probes for immunofluorescence staining.

| **Antibodies for MOUSE: immunohistochemistry** | | | | | |
|---|---|---|---|---|---|
| **Antibody target** | **Supplier** | **Product number** | **Dilution** | **Concentration** | **Webpage** |
| PanCK | Abcam | ab9377 | 1/200 | 0.2ug/ml | https://www.abcam.com/wide-spectrum-cytokeratin-antibody-ab9377.html |
| aSMA | Sigma Aldrich | A5228 | | 0.4 ug/mL | https://www.sigmaaldrich.com/GB/en/product/sigma/a5228 |
| DAPI | Fisher Scientific | D1306 | 1/2000 | | https://www.thermofisher.com/order/catalog/product/D1306#/D1306 |
| CC3 | Cell Signalling Technology | 9661 | 1/100 | 0.52ug/ml | https://www.cellsignal.co.uk/products/primary-antibodies/cleaved-caspase-3-asp175-antibody/9661?_=1628253333558&Ntt=9661&tahead=true |
| CD8 | Thermofisher | 14-0808-82 | 1/100 | 5ug/ml | https://www.thermofisher.com/antibody/product/CD8a-Antibody-clone-4SM15-Monoclonal/14-0808-82 |
| CD31 | Abcam | ab56299 | 1/200 | 0.03ug/ml | https://www.abcam.com/cd31-antibody-rm0032-1d12-bsa-and-azide-free-ab56299.html |
| CD45 | BD Pharmagen | 550539 | 1/100 | 0.62ug/ml | https://www.bdbiosciences.com/en-us/products/reagents/flow-cytometry-reagents/research-reagents/single-color-antibodies-ruo/purified-rat-anti-mouse-cd45.550539 |
| Ki67 | Bethyl | IHC00375 | 1/200 | 1.25ug/ml | https://www.bethyl.com/product/IHC-00375/Ki-67+IHC+Antibody |
| SLUG | Cell Signalling Technology | 9585 | 1/200 | 1.75ug/ml | https://www.cellsignal.co.uk/products/primary-antibodies/slug-c19g7-rabbit-mab/9585 |
| SNAIL | Thermofisher | MA5-14801 | 1/100 | | https://www.thermofisher.com/antibody/product/SNAIL-Antibody-clone-F-31-8-Monoclonal/MA5-14801 |
| Picosirius Red | Abcam | ab150681 | | Kit | https://www.abcam.com/picro-sirius-red-stain-kit-connective-tissue-stain-ab150681.html |
| H + E | Genta | gills I + eosin | | | |

| **Antibodies for MOUSE: immnuoblotting** | | | | | |
|---|---|---|---|---|---|
| **Antibody target** | **Supplier** | **Product number** | **Dilution** | **Host** | **Webpage** |
| SLUG | CST | 9585S | 1/1000 | rabbit | https://www.cellsignal.co.uk/products/primary-antibodies/slug-c19g7-rabbit-mab/9585 |
| SNAIL | CST | 3879S | 1/1000 | rabbit | https://www.cellsignal.co.uk/products/primary-antibodies/snail-c15d3-rabbit-mab/3879 |
| E-CAD | CST | 3195S | 1/1000 | rabbit | https://www.cellsignal.co.uk/products/primary-antibodies/e-cadherin-24e10-rabbit-mab/3195 |
| pSTAT3 | CST | 9145S | 1/1000 | rabbit | https://www.cellsignal.co.uk/products/primary-antibodies/phospho-stat3-tyr705-d3a7-xp-rabbit-mab/9145 |
| beta-actin | Abcam | ab6276 | 1/5000 | mouse | https://www.abcam.com/beta-actin-antibody-ac-15-ab6276.html |
| **Secondary antibodies:** | **Supplier** | **Product number** | **Dilution** | | **Webpage** |
| Anti-mouse IgG (H+L) (DyLight™ 680 Conjugate) | CST | 5470S | 1/15,000 | goat | https://www.cellsignal.co.uk/products/secondary-antibodies/anti-mouse-igg-h-l-dylight-680-conjugate/5470?site-search-type=Products&N=4294956287&Ntt=5470s&fromPage=plp&_requestid=2210153 |
| Anti-rabbit IgG (H+L) (DyLight™ 800 4x PEG Conjugate) | CST | 5151S | 1/15,000 | goat | https://www.cellsignal.co.uk/products/secondary-antibodies/anti-rabbit-igg-h-l-dylight-800-4x-peg-conjugate/5151?site-search-type=Products&N=4294956287&Ntt=5151s&fromPage=plp&_requestid=2210163 |

| **Antibodies for HUMAN: immunofluoresence** | | | | | |
|---|---|---|---|---|---|
| **Antibody target** | **Supplier** | **Product number** | **Dilution** | **Concentration** | **Webpage** |
| PanCK | Abcam | ab9377 | 1/200 | 0.2ug/ml | https://www.abcam.com/wide-spectrum-cytokeratin-antibody-ab9377.html |
| VIM | Agilent | M0725 | 1/100 | | https://www.agilent.com/store/en_US/Prod-M072529-2/M072529-2 |
| **Secondary antibodies:** | **Supplier** | **Product number** | **Dilution** | | **Webpage** |
| Donkey anti-Mouse IgG (H+L) Highly Cross-Adsorbed Secondary Antibody, Alexa Fluor 647 | Invitrogen | A-31571 | 1/400 | | https://www.thermofisher.com/antibody/product/Donkey-anti-Mouse-IgG-H-L-Highly-Cross-Adsorbed-Secondary-Antibody-Polyclonal/A-31571 |
| Donkey anti-Rabbit IgG (H+L) Highly Cross-Adsorbed Secondary Antibody, Alexa Fluor 488 | Invitrogen | A-21206 | 1/400 | | https://www.thermofisher.com/antibody/product/Donkey-anti-Rabbit-IgG-H-L-Highly-Cross-Adsorbed-Secondary-Antibody-Polyclonal/A-21206 |
| Opal 570 | AKOYA | FP1488001kt | 1/200 | | https://my.akoyabio.com/ccrz__ProductDetails?sku=FP1488001KT&cclcl=en_US |

| ***In situ* hybridisation probes** | | | | | |
|---|---|---|---|---|---|
| **Target** | **Supplier** | **Product number** | **Dilution** | | **Webpage** |
| RNAscope® 2.5 LS Probe. *Homo sapiens* oncostatin M receptor (*OSMR*) transcript variant 4 mRNA | Bio-Techne Ltd | 537128 | Ready to use | | https://acdbio.com/rnascope-25-hd-assay%E2%80%94red?_ga=2.110814278.2121125462.1628253100-2047742623.1628253100 |

## Supplementary Table 6. Antibodies used for FACS.

| Target | Fluorophore | Clone | Manufacturer | Product number |
|---|---|---|---|---|
| Fc block | - | 2.4G2 | BD Biosciences | 558636 |
| Live/dead | Near IR | | Thermo Fisher Scientific | L10119 |
| EpCAM | FITC | G8.8 | Biolegend | 118208 |
| CD45 | FITC | 30-F11 | Biolegend | 103108 |
| CD31 | FITC | MED13.3 | Biolegend | 102506 |
| PDPN | APC | 8.1.1 | Biolegend | 127410 |
| CD90 | PE | G7 | Abcam | ab24904 |

## Supplementary Table 7. Genes, primer sequences and probes used for BioMark HD qPCR, with annotations as iCAF score contributors or housekeeping genes as appropriate. F, forward; R, reverse.

| Gene | Sequence | Probe number | iCAF score | Housekeeping genes |
|---|---|---|---|---|
| Acta2_F | ctctcttccagccatctttcat | 58 | | |
| Acta2_R | tataggtggtttcgtggatgc | 58 | | |
| C3_F | gtgggagaagttcggcatag | 81 | Y | |
| C3_R | gagctgggctgtttgaagg | 81 | Y | |
| Ccl2_F | catccacgtgttggctca | 62 | Y | |
| Ccl2_R | gatcatcttgctggtgaatgagt | 62 | Y | |
| Ccl5_F | tgcagaggactctgagacagc | 110 | Y | |
| Ccl5_R | gagtggtgtccgagccata | 110 | Y | |
| Ccl7_F | ttctgtgcctgctgctcata | 89 | Y | |
| Ccl7_R | ttgacatagcagcatgtggat | 89 | Y | |
| Ccnd1_F | caacgcactttctttccagag | 16 | | |
| Ccnd1_R | agggcttcaatctgttcctg | 16 | | |
| Ccnd2_F | cggatccaagtctgtggaag | 77 | | |
| Ccnd2_R | aacatcccgcacgtctgta | 77 | | |
| Cd14_F | aaagaaactgaagcctttctcg | 26 | Y | |
| Cd14_R | agcaacaagccaagcacac | 26 | Y | |
| Cdc42bpg_F | caagtcacgaagccacgag | 20 | | |
| Cdc42bpg_R | aacactgttaggtagggtgctgt | 20 | | |
| Chuk_F | gccagggagacttgatgg | 50 | | |
| Chuk_R | gaggtctgtgctttagctgctt | 50 | | |
| Col11a1_F | aaggacttcctggtccacaa | 2 | | |
| Col11a1_R | caagccctggttttccttg | 2 | | |
| Col12a1_F | actcagatggagaggggaatc | 20 | | |
| Col12a1_R | ccttggtccacttcttggaa | 20 | | |
| Col14a1_F | atgtggattccggtctatgg | 79 | | |
| Col14a1_R | agagtcctgttcttctttgaggtc | 79 | | |
| Col15a_F | ccagggctaaaaggagaaca | 1 | | |
| Col15a_R | ggacgtccccagtcaaga | 1 | | |
| Col15a1_F | ccagggctaaaaggagaaca | 1 | | |
| Col15a1_R | ggacgtccccagtcaaga | 1 | | |
| Col1a1_F | caggcaagcctggtgaac | 80 | | |
| Col1a1_R | aacctctctcgcctcttgc | 80 | | |
| Col3a1_F | tcccctggaatctgtgaatc | 49 | | |
| Col3a1_R | tgagtcgaattggggagaat | 49 | | |
| Col4a2_F | gaaagcgcacaaccagga | 51 | | |
| Col4a2_R | tacaggaaaggcatggtgct | 51 | | |
| Col4a5_F | ccaggaccaaaaggaatcag | 79 | | |
| Col4a5_R | cccaggaagacctggtgtc | 79 | | |
| Col4a6_F | cctggaccctcaggcttaat | 1 | | |
| Col4a6_R | tccattcagtccatgtaaacca | 1 | | |
| Col6a1_F | ttgagcaaggatgagctggt | 5 | | |
| Col6a1_R | gtccacgtgctcttgcatc | 5 | | |
| Col6a2_F | cctatggagagtgctacaaggtg | 8 | | |
| Col6a2_F | tctcctggttcacccatgtt | 8 | | |
| Csf1_F | gggggcctcctgttctac | 25 | | |
| Csf1_R | cccacagaagaatccaatgtc | 25 | | |
| Cxcl1_F | gactccagccacactccaac | 83 | Y | |
| Cxcl1_R | tgacagcgcagctcattg | 83 | Y | |
| Cxcl10_F | gctgccgtcattttctgc | 3 | | |
| Cxcl10_R | tctcactggcccgtcatc | 3 | | |
| Cxcl12_F | ccaaactgtgcccttcagat | 41 | | |
| Cxcl12_R | atttcgggtcaatgcacact | 41 | | |
| Cxcl16_F | gtgggtccgtgaactagtgg | 103 | | |
| Cxcl16_R | gcaaatgtttttggtggtga | 103 | | |
| Cxcl9_RT_F | ccatgaagtccgctgttctt | 105 | | |
| Cxcl9_RT_R | gcatcgtgcattccttatca | 105 | | |
| Dcn_F | gagggaactccacttggaca | 3 | Y | |
| Dcn_R | ttgttgttgtgaaggtagacgac | 3 | Y | |
| Des_F | gcgtgacaacctgatagacg | 110 | | |
| Des_R | tggatttcctcctgtagtttgg | 110 | | |
| Egfr_F | gtgatccaagctgtcccaat | 3 | | |
| Egfr_R | ttggtcaatttctggcagttc | 3 | | |
| Fabp7_F | aaccagcatagatgacagaaactg | 58 | | |
| Fabp7_R | acttctgcacatgaatgagctt | 58 | | |
| Fgf2_F | cggctctactgcaagaacg | 4 | | |
| Fgf2_R | tgcttggagttgtagtttgacg | 4 | | |
| Fn1_F | cggagagagtgcccctacta | 52 | | |
| Fn1_R | cgatattggtgaatcgcaga | 52 | | |
| Gapdh_F | gggttcctataaatacggactgc | 52 | | Y |
| Gapdh_R | ccattttgtctacgggacga | 52 | | Y |
| Gne_F | gagaggaagttcggccaag | 2 | | |
| Gne_R | atcagttcgtgctggtggat | 2 | | |
| Grem1_F | acccacggaagtgacagaat | 63 | | |
| Grem1_R | ccctcagctgttggcagtag | 63 | | |
| Has1_F | gcatgggctatgctaccaa | 50 | Y | |
| Has1_R | tcaaccaacgaaggaaggag | 50 | Y | |
| Has2_F | tgagcaggagctgaacaaga | 101 | Y | |
| Has2_R | gccaacaatataagcagctgtg | 101 | Y | |
| Hgf_F | cacccccttgggagtattgtg | 100 | | |
| Hgf_R | gggacatcagtctcattcacag | 100 | | |
| Icam1_F | caggagcctccggacttt | 11 | | |
| Icam1_R | gaggtcaggggtgtcgag | 11 | | |
| Igf1_F | caaaagcagcccgctcta | 104 | | |
| Igf1_R | tgcacttcctctacttgtgttctt | 104 | | |
| Igfbp7_F | tgccctccatgaaataccac | 110 | | |
| Igfbp7_R | ggctgtctgagagcaccttt | 110 | | |
| Il1r1_F | ttgacatagtgctttggtacagg | 15 | | |
| Il1r1_R | tcgtatgtctttccatctgaagc | 15 | | |
| Il4ra_F | ttaccacagcccctaagcag | 55 | Y | |
| Il4ra_R | ccacaggaggtagatcttgga | 55 | Y | |
| Il6_F | gctaccaaactggatataatcagga | 6 | Y | |
| Il6_R | ccaggtagctatggtactccagaa | 6 | Y | |
| Il6ra_F | atcctctggaaccccacac | 53 | | |
| Il6ra_R | gaactttcgtactgatcctcgtg | 53 | | |
| Il6st_F | tgcttagccttcctcctgac | 3 | | |

| | | | | |
|---|---|---|---|---|
| Il6st_R | gaacattaggccagatgtgtttt | 3 | | |
| Itga5_F | tcacagggcctggagttg | 1 | | |
| Itga5_R | tgtgttcctgaggcagtagaac | 1 | | |
| Itga6_F | gcggctactttcactaaggact | 45 | | |
| Itga6_R | ttcttttgttctacacggacga | 45 | | |
| Itgav_F | ggtgtggatcgagctgtctt | 21 | | |
| Itgav_R | caaggccagcatttacagtg | 21 | | |
| Jak1_F | ggagtactacacagtcaaggacga | 26 | | |
| Jak1_R | aaacattccggagcgtacc | 26 | | |
| Jak2_F | aagattgccaaggccaga | 105 | Y | |
| Jak2_R | ctcacattgttgttccagcac | 105 | Y | |
| Lif_F | tacttgaaggggaccagagg | 1 | | |
| Lif_R | ccctgacctcaagttctgct | 1 | | |
| Lifra_F | gggagtttagagtgctgctca | 34 | | |
| Lifra_R | gtcagtggtccttgagaatgg | 34 | | |
| Lox_F | caggctgcacaatttcacc | 48 | | |
| Lox_R | caaacaccaggtacggcttt | 48 | | |
| Ly6a_F | cctaccctgatggagtctgtgt | 16 | | |
| Ly6a_R | ggcagatgggtaagcaaaga | 16 | | |
| Mcam_F | caaactggtgtgcgtcttctt | 27 | | |
| Mcam_R | cttttcctctcctggcacac | 27 | | |
| Met_F | gctctggaggacaagaccac | 76 | | |
| Met_R | ttctgctacaccgtcagcttt | 76 | | |
| Mmp11_F | tggatgcagcttttgaggat | 81 | | |
| Mmp11_R | gcctaggactggcttctcac | 81 | | |
| Mmp13_F | gccagaacttcccaaccat | 89 | | |
| Mmp13_R | tcagagcccagaattttctcc | 89 | | |
| Mmp3_F | tgcagctctactttgttctttga | 7 | Y | |
| Mmp3_R | agagatttgcgccaaaagtg | 7 | Y | |
| Mt2_F | ccgatctctcgtcgatcttc | 70 | Y | |
| Mt2_R | caggagcaggatccatcg | 70 | Y | |
| Myc_F | cctagtgctgcatgaggagac | 77 | | |
| Myc_R | tccacagacaccacatcaattt | 77 | | |
| Nfkb1_F | gaggagaccggcaactca | 27 | Y | |
| Nfkb1_R | gtccatctccttggtctgct | 27 | Y | |
| Nos2_F | ctttgccacggacgagac | 13 | Y | |
| Nos2_R | tcattgtactctgagggctgac | 13 | Y | |
| Nt5e_F | atgaacatcctgggctacga | 48 | | |
| Nt5e_R | gtccttccacaccgttatcaa | 48 | | |
| Pdgfra_F | aagacctgggcaagaggaac | 100 | | |
| Pdgfra_R | gaacctgtctcgatggcact | 100 | | |
| Pdgfrb_F | tcaagctgcaggtcaatgtc | 67 | | |
| Pdgfrb_R | ccattggcagggtgactc | 67 | | |
| Pdk1_F | gttgaaacgtcccgtgct | 20 | | |
| Pdk1_R | gcgtgatatgggcaatcc | 20 | | |
| Pdpn_F | cagtgttgttctgggttttgg | 95 | Y | |
| Pdpn_R | acctggggtcacaatatcatct | 95 | Y | |
| Pgk1_F | tacctgctggctggatgg | 108 | | |
| Pgk1_R | cacagcctcggcatatttct | 108 | | |
| Ppia_F | gccaccctccctaactgc | 103 | | Y |
| Ppia_R | gcgggctcctactagatggt | 103 | | Y |
| Ptgs2_F | gccgtacacatcatttgaagaa | 46 | | |
| Ptgs2_R | tgtcactgtagagggctttcaa | 46 | | |
| S100a4_F | ggagctgcctagcttcctg | 56 | | |
| S100a4_R | tcctggaagtcaacttcattgtc | 56 | | |
| Saa3_F | ATGCTCGGGGGAACTATGAT | 26 | | |
| Saa3_R | ACAGCCTCTCTGGCATCG | 26 | | |
| Sparc_F | gcccctcagcagactgaa | 73 | | |
| Sparc_R | ggttggcacccacaggta | 73 | | |
| Spp1_F | cccggtgaaagtgactgatt | 82 | | |
| Spp1_R | ttcttcagaggacacagcattc | 82 | | |
| Tagln_F | gcccagacaccgaagcta | 12 | | |
| Tagln_R | gtaggatggacccttgttgg | 12 | | |
| Tbp_F | ggcggtttggctaggttt | 107 | | Y |
| Tbp_R | gggttatcttcacacaccatga | 107 | | Y |
| Thbs1_F | ccccaaccttcccaactc | 4 | | |
| Thbs1_R | gggttgtaatggaatggacag | 4 | | |
| Tnc_F | gctggtgcacccagagac | 85 | | |
| Tnc_R | acagttggatgtccccaatc | 85 | | |
| Tnfrsf1a_F | ggaaagtatgtccattctaagaacaa | 84 | | |
| Tnfrsf1a_R | agtcactcaccaagtaggttcctt | 84 | | |
| Tnfrsf1b_F | gaggcccaagggtttcag | 1 | | |
| Tnfrsf1b_R | ggcttccgtgggaagaat | 1 | | |
| Traf2_F | ctgcagagcaccctgtagc | 85 | | |
| Traf2_R | aacttggggcagacctcata | 85 | | |
| Tubb4a_F | gacctatcatggggacagtga | 55 | | Y |
| Tubb4a_R | cggctctgggaacatagttt | 55 | | Y |
| Vcam1_F | tcttacctgtgcgctgtgac | 47 | | |
| Vcam1_R | gacctccacctgggttctct | 47 | | |
| Vcan_F | cactggctgtggatggtg | 62 | Y | |
| Vcan_R | gttgagcagcggcaaagt | 62 | Y | |

## Supplementary Table 8. Cell lines used in this study.

| Annotation | Cell line | Description | Source |
|---|---|---|---|
| MØ | RAW264.7 | Mouse leukemic monocyte/macrophage cell line | ATCC |
| MØ | *where stated* | Bone marrow-derived macrophages isolated from female BL6 mice | In-house CRUK MI |
| PCC | iKRAS1 | Doxycyline inducible *Kras* $^{G12D}$ -expressing mouse PDA cancer cells. Genotype: *p48-Cre; ROSA26-LSL-rtTA-IRES-GFP; TetO-LSL-Kras* $^{G12D}$ *; p53* $^{fl/+}$ | Dr Ronald DePinho |
| PCC1 | 8296 | Primary PDA cell line isolated from a PK mouse | Dr Dieter Saur |
| PCC2 | 8248 | Primary PDA cell line isolated from a PK mouse | Dr Dieter Saur |
| PCC3 | 9091 | Primary PDA cell line isolated from a PK mouse | Dr Dieter Saur |
| PCC4 | KPC47 | Primary PDA cell line isolated from a KPC mouse. BL6KPC-TB32047. | Dr Kris Freese |
| PCC5 | 8442 | Primary PDA cell line isolated from a PK mouse | Dr Dieter Saur |
| PCC6 | 8570 | Primary PDA cell line isolated from a PK mouse | Dr Dieter Saur |
| PCC7 | 9591 | Primary PDA cell line isolated from a PK mouse | Dr Dieter Saur |
| PCC8 | 8661 | Primary PDA cell line isolated from a PK mouse | Dr Dieter Saur |
| PCC9 | 8182 | Primary PDA cell line isolated from a PK mouse | Dr Dieter Saur |
| PCC10 | 8028 | Primary PDA cell line isolated from a PK mouse | Dr Dieter Saur |
| PCC11 | KPC43 | Primary PDA cell line isolated from a KPC mouse. BL6KPC-TB32043. | Dr Kris Freese |
| PCC12 | 8513 | Primary PDA cell line isolated from a PK mouse | Dr Dieter Saur |
| PSC | PSC C2 | SV40-immortalised mouse Pancreatic Stellate Cells, clone 2 | Dr Raul Urrutia |
| 293FT | 293FT | Fast growing an transfectable human embryonal kidney cells transformed with the SV40 large T antigen | ThermoFisher, R70007 |

## Supplementary Table 9. Recombinant proteins, inhibitors and neutralising antibodies used in this study.

| Reagent | Manufacturer | Product number | Concentration |
|---|---|---|---|
| **Recombinant protein** | | | |
| OSM | R&D | 495-MO-025 | 25 ng/ml |
| TNFα | PeproTech | 315-01A | 15 ng/ml |
| IL1α | R&D | 400-ML-005 | 10 ng/ml |
| GM-CSF | PeproTech | 315-03 | 0.1 ng/mL |
| CCL5 | PeproTech | 250-07 | 1 ng/mL |
| CXCL10 | PeproTech | 250-16 | 5 ng/mL |
| LIF | PeproTech | 250-02 | 2 ng/mL |
| CCL2 | PeproTech | 250-10 | 1 ng/mL |
| CXCL5 | R&D | 433-MC-025 | 5 ng/mL |
| CXCL1 | PeproTech | 250-11 | 1 ng/mL |
| TGFβ1 | R&D | 7666-MB-005 | 5 ng/mL |
| IL1β | PeproTech | 401-ML-005 | 1 ng/mL |
| **Inhibitors** | | | |
| IKKβ inhibitor: TPCA-1 | SelleckChem | S2824 | 1 μM |
| JAK2 inhibitor: AZD1480 | SelleckChem | S2162 | 2.5 μM |
| **Neutralising antibodies** | | | |
| IgG antibody (IgG) | eBioscience | 16-4321-85 | 2.5 ng/mL |
| OSM antibody (αOSM) | R&D | AF-495-NA | 2.5 ng/mL |
| TNFα antibody | Sino Biological | 50349-RN023 | 10 ng/mL |
| IL1β antibody | eBioscience | 14-7012-85 | 1 ng/mL |
| IL1α antibody | R&D | MAB400-500 | 3 μg/mL |
| GM-CSF antibody (GM-CSF-415) | R&D | AB-415-NA | 1 μg/mL |
| GM-CSF antibody (GM-CSF-7331) | Thermo Scientific | 16-7331-85 | 1 μg/mL |
