# Supplementary material for: Heterocellular OSM-OSMR signalling reprograms fibroblasts to promote pancreatic cancer growth and metastasis
Source: Nat Commun. 2021 Dec 17;12:7336. doi: 10.1038/s41467-021-27607-8 (PMC8683436; doi:10.1038/s41467-021-27607-8)
Supplement: Supplementary file 2 — Reporting Summary [file 41467_2021_27607_MOESM2_ESM.pdf]

nature research

Corresponding author(s): Claus Jørgensen

Last updated by author(s): 2021/10/19

Reporting Summary

Nature Research wishes to improve the reproducibility of the work that we publish. This form provides structure for consistency and transparency in reporting. For further information on Nature Research policies, see our Editorial Policies and the Editorial Policy Checklist.

Statistics

For all statistical analyses, confirm that the following items are present in the figure legend, table legend, main text, or Methods section.

n/a	Confirmed	
☐	☒	The exact sample size (*n*) for each experimental group/condition, given as a discrete number and unit of measurement
☐	☒	A statement on whether measurements were taken from distinct samples or whether the same sample was measured repeatedly
☐	☒	The statistical test(s) used AND whether they are one- or two-sided<br>*Only common tests should be described solely by name; describe more complex techniques in the Methods section.*
☒	☐	A description of all covariates tested
☐	☒	A description of any assumptions or corrections, such as tests of normality and adjustment for multiple comparisons
☐	☒	A full description of the statistical parameters including central tendency (e.g. means) or other basic estimates (e.g. regression coefficient) AND variation (e.g. standard deviation) or associated estimates of uncertainty (e.g. confidence intervals)
☐	☒	For null hypothesis testing, the test statistic (e.g. *F*, *t*, *r*) with confidence intervals, effect sizes, degrees of freedom and *P* value noted<br>*Give P values as exact values whenever suitable.*
☒	☐	For Bayesian analysis, information on the choice of priors and Markov chain Monte Carlo settings
☒	☐	For hierarchical and complex designs, identification of the appropriate level for tests and full reporting of outcomes
☒	☐	Estimates of effect sizes (e.g. Cohen's *d*, Pearson's *r*), indicating how they were calculated

Our web collection on statistics for biologists contains articles on many of the points above.

Software and code

Policy information about availability of computer code

Data collection	RNAseq was performed on Illumina NextSeq 500 sequencer<br>LUMINEX was measured on MAGPIX<br>Multiplex RT-qPCR was run on Biomark HD<br>FACS sorting of cells was performed on FACSAria III Cell Sorter<br>Ex vivo iRFP imaging of tissues was performed on Li-COR Pearl Imager<br>Mass cytometry was run on Helios Mass Cytometer<br>SILAC-based phosphoproteomics was run on LTQ OrbiTrap Velos Pro
Data analysis	FastQC (http://www.bioinformatics.bbsrc.ac.uk/projects/fastqc/), STAR 2.7 (https://github.com/alexdobin/STAR), DESeq2 Bioconductor Package, Seurat package (v 3.0.2), Survival package (v3.2.3), GSVA (v1.36.0), lmtest (v0.9-37), ggplot2, CIBERSORT R implementation (v1.04) GSEA V4.1.0, Cytofkit2 (V2.0.1), Cytobank (https://www.cytobank.org) , CellPhoneDB (https://github.com/Teichlab/cellphonedb), GEPIA2 (http://gepia2.cancer-pku.cn/#index), cBioPortal (https://www.cbioportal.org/), Proteome Discoverer 2.1, PhosphoSitePlus (v6.5.9.3), MotifAll (http://meme-suite.org/doc/centrimo.html), Cytoscape (v3.7.2), PhosphoPath (3.2) (http://apps.cytoscape.org/apps/phosphopath), HALO (v3.3.2541), BD FACSdiva (v9.0.1), GraphPad Prism7, Flowjo v10, Image Studio Lite (v5.2)

For manuscripts utilizing custom algorithms or software that are central to the research but not yet described in published literature, software must be made available to editors and reviewers. We strongly encourage code deposition in a community repository (e.g. GitHub). See the Nature Research guidelines for submitting code & software for further information.

Data

Policy information about availability of data

All manuscripts must include a data availability statement. This statement should provide the following information, where applicable:

- Accession codes, unique identifiers, or web links for publicly available datasets
- A list of figures that have associated raw data
- A description of any restrictions on data availability

All original source data are freely available: RNAseq data have been deposited into the Gene Expression Omnibus (GEO) database under accession number GSE161359 (https://www.ncbi.nlm.nih.gov/geo/query/acc.cgi?acc=GSE161359). Publicly available scRNAseq datasets of human, KPC and KPP PDA were acquired from NCBI dbGaP (phs001840.v1.p1, https://www.ncbi.nlm.nih.gov/gap/advanced_search/?TERM=phs001840.v1.p1), GEO (GSE129455, https://www.ncbi.nlm.nih.gov/geo/query/acc.cgi?acc=GSE129455) and ArrayExpress (E-MTAB-8483, https://www.ebi.ac.uk/arrayexpress/experiments/E-MTAB-8483/), respectively. Publicly available RNAseq dataset of my/i/apCAFs were acquired from GEO (GSE93313, https://www.ncbi.nlm.nih.gov/geo/query/acc.cgi?acc=GSE93313). Patient data from TCGA were downloaded from cBioPortal (https://www.cbioportal.org/). Phosphoproteomics mass spectrometry datasets have been deposited to the ProteomeXchange Consortium via the PRIDE partner repository with the dataset identifier PXD022484. Source data are provided with this paper and available online. The remaining data are available within the Article, Supplementary Information or Source Data file.

Field-specific reporting

Please select the one below that is the best fit for your research. If you are not sure, read the appropriate sections before making your selection.

[x] Life sciences [ ] Behavioural & social sciences [ ] Ecological, evolutionary & environmental sciences

For a reference copy of the document with all sections, see nature.com/documents/nr-reporting-summary-flat.pdf

Life sciences study design

All studies must disclose on these points even when the disclosure is negative.

Sample size	No statistical measures were conducted to predetermine the sample size. All sample size, statistical tests and p-values are indicated in the figure legends and described in materials and methods.<br>For animal experiments, the 3R guidelines were closely followed to reduce the number of animals used in this study while retaining statistical power.
Data exclusions	No data were excluded from analysis
Replication	Replicates and independent experiments were performed to verify reproducibility of the assay. All attempts at replication were successful.
Randomization	Samples were prepared, treated, processed and analysed in random order.
Blinding	Investigators were blinded during all histological analyses and mouse injections. The remaining experiments were not blinded as the investigators who set up the experiments analysed the data, which is incompatible with blinding.

Reporting for specific materials, systems and methods

We require information from authors about some types of materials, experimental systems and methods used in many studies. Here, indicate whether each material, system or method listed is relevant to your study. If you are not sure if a list item applies to your research, read the appropriate section before selecting a response.

Materials & experimental systems

n/a	Involved in the study	
[ ]	[x]	Antibodies
[ ]	[x]	Eukaryotic cell lines
[x]	[ ]	Palaeontology and archaeology
[ ]	[x]	Animals and other organisms
[ ]	[x]	Human research participants
[x]	[ ]	Clinical data
[x]	[ ]	Dual use research of concern

Methods

n/a	Involved in the study	
[x]	[ ]	ChIP-seq
[ ]	[x]	Flow cytometry
[x]	[ ]	MRI-based neuroimaging

Antibodies

Antibodies used	Fc block,BD Biosciences,558636<br>CD44,Biolegend,103002<br>MCAM,Fluidigm,3141016B

ITGA5,Biolegend,103801
CD81,Biolegend,104902
CD87,Thermo Fisher,MA5-23853, clone 109801
PE (ITGA1),Fluidigm,3145006B
ITGAV,Biolegend,104102
ITGA2,Biolegend,103501
PDGFRA,Fluidigm,3148018B
PDPN,Biolegend,127402
CD24,Fluidigm,3150009B
PDGFRB,Fluidigm,3151017B
ICAM1,Biolegend,116102
CD73,Biolegend,127202
ITGB3,Biolegend,104302
CD34,Biolegend,119302
ITGA6,Biolegend,313602
Biotin (CD105),Fluidigm,3165012B
CD14,Biolegend,123302
CD47,Biolegend,151002
GFP,Biolegend,338002
CD38,Fluidigm,3171007B
ITGB1,Biolegend,102202
VCAM1,Biolegend,105702
CD45,Fluidigm,3175010B
APC (CD90),Fluidigm,3176007B
Donkey anti-Mouse IgG (H+L) Highly Cross-Adsorbed Secondary Antibody, Alexa Fluor 647, Invitrogen, A-31571
Donkey anti-Rabbit IgG (H+L) Highly Cross-Adsorbed Secondary Antibody, Alexa Fluor 488, Invitrogen, A-21206
Opal 570, AKOYA, FP1488001kt
MHCII,Fluidigm,3209006B
CD47,Biolegend,127602
CD64 ,X54-5/7.1 ,Fluidigm,3151012B
CD16/CD32 ,93,Biolegend,101302
CD11b ,M1/70 ,Biolegend,101202
PDCA1,927,Biolegend,127002
CD68,FA-11 ,Biolegend,137002
Ly6G,1A8 ,Fluidigm,3141008B
Siglec-F,E50-2440 ,BD Biosciences,552125
PD-L1,10F.9G2 ,Biolegend,124302
F4/80,BM8 ,Fluidigm,3146008B
MHCI ,28-14-8 ,Biolegend,114502
CD3e ,17A2 ,BD Biosciences,555273
CD19,6D5 ,Fluidigm,3149002B
CD1d,1B1 ,Biolegend,123502
CD11c ,N418 ,Biolegend,117302
CD63 ,NVG-2 ,Biolegend,143902
XCR1,ZET ,Biolegend,148202
TCRb ,H57-597 ,Biolegend,109201
CX3CR1,SA011F11 ,Biolegend,149002
CXCR2,SA044G4 ,Biolegend,149302
CSF1R,AFS98 ,Biolegend,135502
CD40,HM40-3 ,Biolegend,102902
CD103,20000000,Biolegend,121402
PD-L2,TY25 ,Biolegend,107202
VISTA ,MIH63 ,Biolegend,150202
SIRPa,P84 ,Biolegend,144002
IL-4Ra,I015F8 ,Biolegend,144802
CD206,C086C2 ,Fluidigm,3169021B
CD49b ,HMa2 ,Fluidigm,3170008B
CD80,16-10A1 ,Fluidigm,3171008B
CD86,GL1 ,Fluidigm,3172016B
NKp46 ,29A1.4 ,Biolegend,137602
Ly6C,HK1.4 ,Biolegend,128002
CD24,M1/69 ,Biolegend,101802
SLAM F6,TC15-12F12.2 ,Biolegend,115902
CC3,D3E9 ,Cell Signaling Technology,9579
Ki67,So1A15 ,Thermo Fisher,14-5698-82
Galectin-9,9M1-3 ,Thermo Fisher,16-9116-85
CD63,NVG-2 ,Biolegend,143902
iNOS,CXNFT ,Fluidigm,3161011B

TCRb ,H57-597 ,Biolegend,109202
GITR,DTA1 ,Fluidigm,3143019B
CD44,IM7 ,Biolegend,103002
CD69,H1.2F3 ,Fluidigm,3145005B
TIGIT,1G9 ,Biolegend,142102
4-1BB,17B5 ,Biolegend,106107
CD27,LG.3A10 ,Fluidigm,3150017B
LAG3,C9B7W ,Biolegend,125202
CD8a,53-6.7 ,Fluidigm,3153012B
CTLA4 ,UC10-4B9 ,Fluidigm,3154008B
CD4,RM4-5 ,Biolegend,100506
SLAM F7,TC15-12F12.2 ,Biolegend,115902
PD-1,29F.1A12 ,Fluidigm,3159024B
CD62L,MEL-14 ,Fluidigm,3160008B
TIM3,RMT3-23 ,Fluidigm,3162029B
CD49b ,HMa2 ,Biolegend,103501
CD103,20000000,Biolegend,121402
KLRG1,2F1 ,BD Biosciences,562190
ICOS,C398.4A ,Fluidigm,3168024B
CD39,24DMS1 ,Thermo Fisher,14-0391-82
CD25,PC61 ,Biolegend,102002
CD127,A7R34 ,Fluidigm,3174013B
TCRgd,UC7-13D5 ,Biolegend,107502
CD73,TY/11.8 ,Biolegend,127202
SLAM F6,TC15-12F12.2 ,Biolegend,115902
GATA3,L50-823 ,BD Biosciences,558686
GZMB,GB11 ,Thermo Fisher,MA1-80734
TCF1,C63D9 ,Cell Signaling Technology,2203
EOMES,Dan11mag ,Thermo Fisher,14-4875-82
TBET,4B10 ,Fluidigm,3160010B
FOXP3,FJK-16s ,Fluidigm,3165024A
TOX,REA473,Miltenyi Biotech,130-126-455
RUNX3,R3-5G4,Biolegend,697902
RORgT,Q31-378,BD Biosciences,562663
Pan Cytokeratin,pAb,Abcam,9377,2ug/mL
aSMA,1A4,Sigma Aldrich,A5228,0.4 ug/mL
Live/dead,Near IR,,Thermo Fisher Scientific,L10119
EpCAM,FITC,G8.8,Biolegend,118208
CD45,FITC,30-F11,Biolegend,103108
CD31,FITC,MED13.3,Biolegend,102506
PDPN,APC,8.1.1,Biolegend,127410
CD90,PE,G7,Abcam,ab24904
Donkey anti-Mouse IgG (H+L) Highly Cross-Adsorbed Secondary Antibody, Alexa Fluor 647, Invitrogen, A-31571
Donkey anti-Rabbit IgG (H+L) Highly Cross-Adsorbed Secondary Antibody, Alexa Fluor 488, Invitrogen, A-21206
Opal 570, AKOYA, FP1488001kt

Validation

Fc block (https://www.bdbiosciences.com/us/applications/research/b-cell-research/surface-markers/mouse/apc-rat-anti-mouse-cd16cd32-fc-iiiii-receptor-24g2/p/558636)
CD44 (https://www.biolegend.com/en-us/products/purified-anti-mouse-human-cd44-antibody-318)
MCAM (https://www.fluidigm.com/reagents/proteomics/3141016b-antimouse-cd146--me-9f1--141pr--100tests)
ITGA5 (https://www.biolegend.com/en-us/products/purified-anti-mouse-cd49e-antibody-169)
CD81 (https://www.biolegend.com/en-us/products/purified-anti-mouse-rat-cd81-antibody-239)
CD87 (https://www.thermofisher.com/antibody/product/uPAR-Antibody-clone-109801-Monoclonal/MA5-23853)
PE (ITGA1) (https://www.fluidigm.com/reagents/proteomics/3145006b-antipe--pe001--145nd--100tests)
ITGAV (https://www.biolegend.com/en-us/products/purified-anti-mouse-cd51-antibody-489)
ITGA2 (https://www.biolegend.com/en-us/products/purified-anti-mouse-cd49b-antibody-300)
PDGFRA (https://www.fluidigm.com/reagents/proteomics/3148018b-antimouse-cd140a--apa5--148nd--100tests)
PDPN (https://www.biolegend.com/en-us/products/purified-anti-mouse-podoplanin-antibody-4749)
CD24 (https://www.fluidigm.com/reagents/proteomics/3150009b-antimouse-cd24--m1-69--150nd--100tests)
PDGFRB (https://www.fluidigm.com/reagents/proteomics/3151017b-antimouse-cd140b--apb5--151eu--100tests)
ICAM1 (https://www.biolegend.com/en-us/products/purified-anti-mouse-cd54-antibody-1677)
CD73 (https://www.biolegend.com/en-us/products/purified-anti-mouse-cd73-antibody-4679)
ITGB3 (https://www.biolegend.com/en-us/products/purified-anti-mouse-rat-cd61-antibody-82)
CD34 (https://www.biolegend.com/en-us/products/purified-anti-mouse-cd34-antibody-2601)
ITGA6 (https://www.biolegend.com/en-us/products/purified-anti-human-mouse-cd49f-antibody-2604)
Biotin (CD105) (https://www.fluidigm.com/reagents/proteomics/3165012b-antibiotin--1d4-c5--165ho--100tests)
CD14 (https://www.biolegend.com/en-us/products/purified-anti-mouse-cd14-antibody-4324)
CD47 (https://www.biolegend.com/en-us/products/purified-anti-mouse-cd74-clip-antibody-12565)

GFP (https://www.biolegend.com/en-us/products/purified-anti-gfp-antibody-5478)
CD38 (https://www.fluidigm.com/reagents/proteomics/3171007b-antimouse-cd38--90--171yb--100tests)
ITGB1 (https://www.biolegend.com/en-us/products/purified-anti-mouse-rat-cd29-antibody-306)
VCAM1 (https://www.biolegend.com/en-us/products/purified-anti-mouse-cd106-antibody-139)
CD45 (https://www.fluidigm.com/reagents/proteomics/3175010b-antimouse-cd45--30-f11--175lu--100tests)
APC (CD90) (https://www.fluidigm.com/reagents/proteomics/3176007b-antiapc--apc003--176yb--100tests)
MHCII (https://www.fluidigm.com/reagents/proteomics/3209006b-anti-mouse-i-a-i-e-m5-114.15.2-209bi-100-tests)
CD47 (https://www.biolegend.com/en-us/products/purified-anti-mouse-ly-6g-antibody-4767)
CD64 (https://www.fluidigm.com/reagents/proteomics/3151012b-antimouse-cd64--x54-5-7-1--151eu--100tests)
CD16/CD32 (https://www.biolegend.com/en-us/products/purified-anti-mouse-cd16-32-antibody-190)
CD11b (https://www.biolegend.com/en-us/products/purified-anti-mouse-human-cd11b-antibody-351)
PDCA1 (https://www.biolegend.com/en-us/products/purified-anti-mouse-cd317-bst2-pdca-1-antibody-6229)
CD68 (https://www.biolegend.com/en-us/products/purified-anti-mouse-cd68-antibody-6421)
Ly6G (https://www.fluidigm.com/reagents/proteomics/3141008b-antimouse-ly-6g--1a8--141pr--100tests)
Siglec-F (https://www.bdbiosciences.com/us/applications/research/b-cell-research/surface-markers/mouse/purified-rat-anti-mouse-siglec-f-e50-2440/p/552125)
PD-L1 (https://www.biolegend.com/en-us/products/purified-anti-mouse-cd274-b7-h1-pd-l1-antibody-4481)
F4/80 (https://www.fluidigm.com/reagents/proteomics/3146008b-antimouse-f4-80--bm8--146nd--100tests)
MHCI (https://www.biolegend.com/en-us/products/purified-anti-mouse-h-2ld-h-2db-antibody-1687)
CD3e (https://www.bdbiosciences.com/us/applications/research/t-cell-immunology/th-1-cells/surface-markers/mouse/purified-rat-anti-mouse-cd3-molecular-complex-17a2/p/555273)
CD19 (https://www.fluidigm.com/reagents/proteomics/3149002b-antimouse-cd19--6d5--149sm--100tests)
CD1d (https://www.biolegend.com/en-us/products/purified-anti-mouse-cd1d-cd1-1-ly-38-antibody-4316)
CD11c (https://www.biolegend.com/en-us/products/purified-anti-mouse-cd11c-antibody-1817)
CD63 (https://www.biolegend.com/en-us/products/purified-anti-mouse-cd63-antibody-7813)
XCR1 (https://www.biolegend.com/en-us/products/purified-anti-mouse-rat-xcr1-antibody-10184)
TCRb (https://www.biolegend.com/en-us/products/purified-anti-mouse-tcr-beta-chain-antibody-274)
CX3CR1 (https://www.biolegend.com/en-us/products/purified-anti-mouse-cx3cr1-antibody-10373)
CXCR2 (https://www.biolegend.com/en-us/products/purified-anti-mouse-cd182-cxcr2-antibody-11708)
CSF1R (https://www.biolegend.com/en-us/products/purified-anti-mouse-cd115-csf-1r-antibody-6214)
CD40 (https://www.biolegend.com/en-us/products/purified-anti-mouse-cd40-antibody-290)
CD103 (https://www.biolegend.com/en-us/products/purified-anti-mouse-cd103-antibody-3572)
PD-L2 (https://www.biolegend.com/en-us/products/purified-anti-mouse-cd273-b7-dc-pd-l2-antibody-2545)
VISTA (https://www.biolegend.com/en-us/products/purified-anti-mouse-vista-pd-1h-antibody-11983)
SIRPa (https://www.biolegend.com/en-us/products/purified-anti-mouse-cd172a-sirpalpha-antibody-7827)
IL-4Ra (https://www.biolegend.com/en-us/products/purified-anti-mouse-cd124-il-4ralpha-antibody-8179)
CD206 (https://www.fluidigm.com/reagents/proteomics/3169021b-anti-mouse-cd206-mmr--c068c2--169tm--100-tests)
CD49b (https://www.fluidigm.com/reagents/proteomics/3170008b-antimouse-cd49b--hma2--170er--100tests)
CD80 (https://www.fluidigm.com/reagents/proteomics/3171008b-antimouse-cd80--16-10a1--171yb--100tests)
CD86 (https://www.fluidigm.com/reagents/proteomics/3172016b-antimouse-cd86--gl1--172yb--100tests)
NKp46 (https://www.biolegend.com/en-us/products/purified-anti-mouse-cd335-nkp46-antibody-6526)
Ly6C (https://www.biolegend.com/en-us/products/purified-anti-mouse-ly-6c-antibody-4894)
CD24 (https://www.biolegend.com/en-us/products/purified-anti-mouse-cd24-antibody-344)
SLAM F6 (https://www.biolegend.com/en-us/products/purified-anti-mouse-cd150-slam-antibody-1368)
CC3 (https://www.cellsignal.co.uk/products/primary-antibodies/cleaved-caspase-3-asp175-d3e9-rabbit-mab/9579)
Ki67 (https://www.thermofisher.com/antibody/product/Ki-67-Antibody-clone-SolA15-Monoclonal/14-5698-82)
Galectin-9 (https://www.thermofisher.com/antibody/product/Galectin-9-Antibody-clone-9M1-3-Monoclonal/16-9116-85)
CD63 (https://www.biolegend.com/en-us/products/purified-anti-mouse-cd63-antibody-7813)
iNOS (https://www.fluidigm.com/reagents/proteomics/3161011b-antimouse-inos--cxnft--161dy--100tests)
TCRb (https://www.biolegend.com/en-us/products/purified-anti-mouse-tcr-beta-chain-antibody-274)
GITR (https://www.fluidigm.com/reagents/proteomics/3143019b-antimouse-cd357-gitr--dta1--143nd--100tests)
CD44 (https://www.biolegend.com/en-us/products/purified-anti-mouse-human-cd44-antibody-318)
CD69 (https://www.fluidigm.com/reagents/proteomics/3145005b-antimouse-cd69--h1-2f3--1
Donkey anti-Mouse IgG (H+L) Highly Cross-Adsorbed Secondary Antibody, Alexa Fluor 647 (https://www.thermofisher.com/antibody/product/Donkey-anti-Mouse-IgG-H-L-Highly-Cross-Adsorbed-Secondary-Antibody-Polyclonal/A-31571)
Donkey anti-Rabbit IgG (H+L) Highly Cross-Adsorbed Secondary Antibody, Alexa Fluor 488 (https://www.thermofisher.com/antibody/product/Donkey-anti-Rabbit-IgG-H-L-Highly-Cross-Adsorbed-Secondary-Antibody-Polyclonal/A-21206)
Opal 570 (https://my.akoyabio.com/ccrz__ProductDetails?sku=FP1488001KT&cclcl=en_US)45nd--100tests)
TIGIT (https://www.biolegend.com/en-us/products/purified-anti-mouse-tigit-vstm3-antibody-7428)
4-1BB (https://www.biolegend.com/en-us/products/leaf-purified-anti-mouse-cd137-antibody-50)
CD27 (https://www.fluidigm.com/reagents/proteomics/3150017b-antihuman-mouse-cd27--lg-3a10--150nd--100tests)
LAG3 (https://www.biolegend.com/en-us/products/purified-anti-mouse-cd223-lag-3-antibody-4482)
CD8a (https://www.fluidigm.com/reagents/proteomics/3153012b-antimouse-cd8a--53-6-7--153eu--100tests)
CTLA4 (https://www.fluidigm.com/reagents/proteomics/3154008b-antimouse-cd152-ctla-4--uc10-4b9--154sm--100tests)
CD4 (https://www.biolegend.com/en-us/products/purified-anti-mouse-cd4-antibody-484)
SLAM F7 (https://www.biolegend.com/en-us/products/purified-anti-mouse-cd150-slam-antibody-1368)
PD-1 (https://www.fluidigm.com/reagents/proteomics/3159024b-antimouse-cd279-pd-1--29f-1a12--159tb--100tests)
CD62L (https://www.fluidigm.com/reagents/proteomics/3160008b-antimouse-cd62l--mel-14--160gd--100tests)
TIM3 (https://www.fluidigm.com/reagents/proteomics/3162029b-antimouse-cd366-tim-3--rmt3-23--162dy--100tests)

CD49b (https://www.biolegend.com/en-us/products/purified-anti-mouse-cd49b-antibody-300)
CD103 (https://www.biolegend.com/en-us/products/purified-anti-mouse-cd103-antibody-3572)
KLRG1 (https://www.bdbiosciences.com/us/reagents/research/antibodies-buffers/immunology-reagents/anti-mouse-antibodies/cell-surface-antigens/purified-nale-hamster-anti-mouse-klrg1-2f1/p/562190)
ICOS (https://www.fluidigm.com/reagents/proteomics/3168024b-anti-cd278-icos-c398.4a-168er%E2%80%94100-tests)
CD39 (https://www.thermofisher.com/antibody/product/CD39-Antibody-clone-24DMS1-Monoclonal/14-0391-82)
CD25 (https://www.biolegend.com/en-us/products/purified-anti-mouse-cd25-antibody-426)
CD127 (https://www.fluidigm.com/reagents/proteomics/3174013b-antimouse-cd127-il7ra--a7r34--174yb--100tests)
TCRgd (https://www.biolegend.com/en-us/products/purified-anti-mouse-tcr-gamma-delta-antibody-520)
CD73 (https://www.biolegend.com/en-us/products/purified-anti-mouse-cd73-antibody-4679)
SLAM F6 (https://www.biolegend.com/en-us/products/purified-anti-mouse-cd150-slam-antibody-1368)
GATA3 (https://www.bdbiosciences.com/us/applications/research/t-cell-immunology/th-2-cells/intracellular-markers/cell-signalling-and-transcription-factors/human/purified-mouse-anti-gata3-l50-823/p/558686)
GZMB (https://www.thermofisher.com/antibody/product/Granzyme-B-Antibody-clone-GB11-Monoclonal/MA1-80734)
TCF1 (https://www.cellsignal.co.uk/products/primary-antibodies/tcf1-tcf7-c63d9-rabbit-mab/2203)
EOMES (https://www.thermofisher.com/antibody/product/EOMES-Antibody-clone-Dan11mag-Monoclonal/14-4875-82)
TBET (https://www.fluidigm.com/reagents/proteomics/3160010b-antihuman-mouse-tbet--4b10--160gd--100tests)
FOXP3 (https://www.fluidigm.com/reagents/proteomics/3165024a-antimouse-rat-foxp3--fjk-16s--165ho--50tests)
TOX (https://www.miltenyibiotec.com/GB-en/products/tox-antibody-anti-human-mouse-reafinity-rea473.html#pure:100-ug-in-100-ul)
RUNX3 (https://www.biolegend.com/en-us/products/purified-anti-runx3-antibody-14654)
RORgT (https://www.bdbiosciences.com/us/applications/research/t-cell-immunology/th17-cells/intracellular-markers/cell-signalling-and-transcription-factors/mouse/purified-mouse-anti-mouse-rort-q31-378/p/562663)
Pan Cytokeratin (https://www.abcam.com/wide-spectrum-cytokeratin-antibody-ab9377.html)
aSMA (https://www.sigmaaldrich.com/catalog/search?term=A5228&interface=All&N=0&mode=match%20partialmax&lang=en&region=GB&focus=product)
Live/dead,Near IR (https://www.thermofisher.com/order/catalog/product/L10119#/L10119)
EpCAM,FITC (https://www.biolegend.com/en-us/products/fitc-anti-mouse-cd326-ep-cam-antibody-4971)
CD45,FITC (https://www.biolegend.com/en-us/products/fitc-anti-mouse-cd45-antibody-99)
CD31,FITC (https://www.biolegend.com/en-us/products/fitc-anti-mouse-cd31-antibody-377)
PDPN,APC (https://www.biolegend.com/en-us/products/apc-anti-mouse-podoplanin-antibody-6656)
CD90,PE (https://www.abcam.com/cd90--thy1-antibody-g7-pe-ab24904.html)
Donkey anti-Mouse IgG (H+L) Highly Cross-Adsorbed Secondary Antibody, Alexa Fluor 647 (https://www.thermofisher.com/antibody/product/Donkey-anti-Mouse-IgG-H-L-Highly-Cross-Adsorbed-Secondary-Antibody-Polyclonal/A-31571)
Donkey anti-Rabbit IgG (H+L) Highly Cross-Adsorbed Secondary Antibody, Alexa Fluor 488 (https://www.thermofisher.com/antibody/product/Donkey-anti-Rabbit-IgG-H-L-Highly-Cross-Adsorbed-Secondary-Antibody-Polyclonal/A-21206)
Opal 570 (https://my.akoyabio.com/ccrz__ProductDetails?sku=FP1488001KT&cclcl=en_US)

Eukaryotic cell lines

Policy information about cell lines

Cell line source(s)	iKRAS cells (denoted as PCC in this study) were obtained from from Dr Ronald DePinho; BL6KPC-TB32043 and BL6KPC-TB32047 (denoted as PCC4 and PCC11 in this study) were obtained from Dr Kris Freese; 8296, 8248, 9091, 8442, 8570, 9591, 8661, 8182, 8028, 8513 (denoted as PCC1, PCC2, PCC3, PCC5, PCC6, PCC7, PCC8, PCC9, PCC10, PCC12, respectively, in this study) are from Dr Dieter Saur. Mouse pancreatic stellate cells (PSCs) were from Dr Raul Urrutia. RAW264.7 is from ATCC. 293FT (ThermoFisher, P70007)
Authentication	RAW264.7 was authenticated by the supplier. 293FT was authenticated by supplier
Mycoplasma contamination	All cells used in experiments were routinely tested free of Mycoplasma. Additionally, cells used in vivo were further tested for Mouse Hepatitis Virus (MHV) and Mycoplasma 5 days prior to the experiment. Commercial cell lines were authenticated by STR profiling, Murine PCC and PSC cell lines were not STR authenticated
Commonly misidentified lines (See ICLAC register)	No commonly misidentified cell lines were used in this study.

Animals and other organisms

Policy information about studies involving animals; ARRIVE guidelines recommended for reporting animal research

Laboratory animals	To establish an immune-competent genetically engineered mouse model (GEMM) with whole-body depletion of Osm (Osm-/-), the cryopreserved sperm from a male C57BL/6 Osm-/- mouse (MMRRC_048921-UCD) was purchased from The KOMP Repository (University of California, Davis, US) and fertilised in vitro with eggs from a female wildtype donor C57BL/6 mouse (purchased from Envigo). The fertilised embryos were implanted into pseudo-pregnant surrogate females. The litters were crossed with age-matched wildtype C57BL/6 mice to produce heterozygotes, which were then crossed further to produce wildtype and Osm-/- breeders. All animals were genotyped and maintained under pathogen-free and ventilated cages with environment enrichment in the Biological Resources Unit at the CRUK Manchester Institute (CRUK MI), and allowed free access to irradiated food and autoclaved water ad libitum in a 12 h light/dark cycle, with room temperature at 21 ± 2 °C and a humidity of 45-65%. 8-12 week old mice were used for orthotopic injections, sex and age were matched as close as possible between Wt and Osm-/- groups.

	To isolated the bone marrow, hind legs were collected from 8-10 week old female C57BL/6 mice, and processed as described in the methods.
Wild animals	No wild animals were used in the study.
Field-collected samples	No field collected samples were used in the study.
Ethics oversight	All animal experiments were were approved by the Animal Welfare and Ethical Review Body (AWERB) of the CRUK MI and performed in accordance with Home Office regulations under the Animals (Scientific Procedures) Act 1986 and under Project License PPL 70/8745. All animal experiments were reported in accordance with Reporting of In Vivo Experiments (ARRIVE) guidelines.

Note that full information on the approval of the study protocol must also be provided in the manuscript.

Human research participants

Policy information about studies involving human research participants

Population characteristics	For ISH and IF analysis of human resected PDA samples were obtained from MCRC biobank.<br>For expression analysis in human PDAC patients, data was obtained from the cancer genome atlas (TCGA) using cBioPortal (https://www.cbioportal.org/, Cerami et al., 2012 & Gao et al., 2013). For population characteristics patient and clinical annotations for the PAAD:TCGA cohort were utilized.
Recruitment	Research samples were obtained from the Manchester Cancer Research Centre (MCRC) Biobank with informed patient consent obtained prior to sample collection.
Ethics oversight	The MCRC Biobank is licensed by the Human Tissue Authority (license number: 30004) and is ethically approved as a research tissue bank by the South Manchester Research Ethics Committee (Ref: 07/H1003/161+5). The role of the MCRC Biobank is to distribute samples. For more information see www.mcrc.manchester.ac.uk/Biobank/Ethics-and-Licensing.

Note that full information on the approval of the study protocol must also be provided in the manuscript.

Flow Cytometry

Plots

Confirm that:

- [x] The axis labels state the marker and fluorochrome used (e.g. CD4-FITC).
- [x] The axis scales are clearly visible. Include numbers along axes only for bottom left plot of group (a 'group' is an analysis of identical markers).
- [x] All plots are contour plots with outliers or pseudocolor plots.
- [x] A numerical value for number of cells or percentage (with statistics) is provided.

Methodology

Sample preparation	CAF isolation from mouse tumours<br>Single-cell dissociation of freshly isolated tumours was performed as described above. Cells were counted for each sample, and CD45+ cells were depleted using mouse CD45 MicroBeads (Miltenyi Biotec, 130-052-301) and LS columns (130-042-401) following manufacturer's recommendation. To discern viable cells, CD45+-depleted samples were stained with LIVE/DEAD Near-IR Dead Cell Stain Kit (LD) (ThermoFisher, L10119) following manufacturer's recommendation. Cells were then washed and pelleted and stained with Fc block (BD Biosciences, 558636) for 5 min. A master mix of lineage and fibroblast marker antibodies (Supplementary Table 2) was then added to each sample and incubated on ice for 30 min. Stained cells were washed in PBS, pelleted and resuspended in FACS Buffer (1 % FBS in PBS) and kept on ice until FACS-isolation. Viable CAFs were sorted on BD FACSAria III Cell Sorter based on LD- CD45- EpCAM- CD31- PDPN+ CD90+ staining profile.<br><br>Fibroblast mass cytometry analysis:<br>Prior to setting up in vitro cultures, PCCs and MØs were labelled with EGFP using a lentivirus approach, as described above. PSC mono-culture (n=3), PCC-PSC (n=3) and PCC-PSC-MØ (n=3) co-cultures were prepared as described above. After 72 h of in vitro cultures, 10 µL of 10 mM 5-iodo-2'-deoxyuridine (IdU) (Sigma Aldrich, 17125) solution in 0.2 M NaOH/water was added directly to the media, gently swirled and the cells incubated at 37 °C for 20 min. Cells were then washed in PBS, dislodged using Accutase Solution and quenched with CSM (Cell Staining Buffer), consisting of 5 mg/ml Bovine Serum Albumin (BSA) (Sigma Aldrich, A3294) in PBS. Cells were filtered through a 70 µm cell strainer (BD Biosciences, 340633). Subsequently, cells were resuspended and counted. Barcoding. 3x106 cells from each culture condition were aliquoted into individual 5 mL polypropylene FACS tubes, pelleted and gentle mixed. A unique pair of lanthanide-conjugated anti-ITGB1 antibody pairs in 50 µL of CSM was then added to each sample. This live-cell barcoding method leveraged the consistently high expression of ITGB1 in fibroblasts, and in this instance a 5-choose-2 scheme (ITGB1-115In, -163Dy, -166Er, -169Tm, -172Yb) was used. Each sample was gently mixed and incubated on ice in the dark for 30 min. Cells from each sample were washed twice with CSM and combined into a 5 mL polypropylene FACS tube. Live/Dead cell staining. The pooled cells were resuspended in 300 µL of ice-cold PBS and vortexed well. 300 µL of 1 µM cisplatin (Fluidigm, 201064) in PBS was added into the cells, and incubated for 1 min. The staining was quenched with CSM, and cells were pelleted and aspirated. Subsequently, 60 µL of 100 U/mL heparin sodium salt (Sigma Aldrich, H3393) solution in PBS and 3 µL Fc block (BD Biosciences, 558636) was added to the cells. The cell mixture was gently mixed and incubated on ice for 5 min. Fluorophore antibody staining. A master mix of fluorophore-conjugated antibodies (Supplementary Table 2) in 150 µL CSM was added, gently mixed and incubated on

ice in the dark for 45 min. Cells were washed with CSM and pelleted. Extracellular antibody staining. A master mix of 6 equivalents of extracellular targeting, metal-conjugated antibodies (Supplementary Table 2) in 150 µL of CSM was added, gently mixed and incubated on ice in the dark for 45 min. Cells were washed twice with CSM. Fixation and permeabilisation. The cell pellet was resuspended in 100 µL of PBS, vortexed and 2 mL of 1x FOXP3 Fixation Buffer from FOXP3 Fixation/ Permeabilization Kit (Thermo Fisher, 00-5523-00) was added, followed by thorough vortexing. After 30 min incubation at RT, 2 mL of 1x FOXP3 Permeabilization Buffer was added and the cells were pelleted. Additional 2 mL of 1x FOXP3 Permeabilization Buffer was added and the cells were pelleted. 60 µL of 100 U/mL heparin sodium salt in PBS and 3 µL of Fc block was added and the sample was mixed by gently rocking. Intracellular antibody staining. After incubating for 5 min at RT in the dark, a master mix of 6 intracellular targeting, metal-conjugated antibodies (Supplementary Table 2) in 150 µL CSM was added. The sample was gently mixed and incubated on ice in the dark for 45 min. Cells were washed twice with CSM. The cell pellet was resuspended in 1 mL of PBS and vortexed well. 2 mL of 4% Paraformaldehyde (PFA) (Thermo Fisher, 28908) in PBS was added. The sample was vortexed and stored overnight at 4 °C in the dark until the mass cytometry analysis following day.

Mass cytometry analysis of immune cells in mouse tumours:
Single-cell dissociation of freshly isolated tumours was performed as described above. Live/Dead cell staining. Each of the disaggregated tumour cell pellet was resuspended in 300 µL of ice-cold PBS, vortexed well and 300 µL of 1 µM 198Pt monoisotopic cisplatin (Fluidigm, 201198) in PBS was added, and incubated for 1 min at RT. The staining was quenched with 20 mL of CSM-E (Cell Staining Buffer – Extracellular) consisting of 5 mg/ml Bovine Serum Albumin (BSA) (Sigma Aldrich, A3294), 0.5% v/v Fetal Bovine Albumin (FBS) (Thermo Fisher, 10270106) and 0.2 mg/mL DNAse1 in PBS. The cells were resuspended and counted. 3x106 cells from each tumour sample were aliquoted into individual 5 mL polypropylene FACS tubes, washed with CSM-E and pelleted. 20 µL of 100 U/mL heparin sodium salt (Sigma Aldrich, H3393) solution in PBS and 1 µL Fc block (BD Biosciences, 558636) was added. The cell mixtures were gently mixed and incubated on ice for 5 min. Extracellular staining. A master mix of extracellular targeting, metal-conjugated antibodies (Supplementary Table 2) in 50 µL of CSM-E was added, gently mixed, incubated on ice in the dark for 45 min. The cells were washed twice with CSM-E. Fixation and permeabilisation. The cell pellet was resuspended in 100 µL of PBS and vortexed and 1 mL of 1x FOXP3 Fixation Buffer from FOXP3 Fixation/Permeabilization Kit (Thermo Fisher, 00-5523-00) was added, followed by thorough vortexing. After 30 min incubation at RT, 2 mL of 1x FOXP3 Permeabilization Buffer was added and the cells pelleted. The cell pellets were resuspended in 1 mL of 10% v/v DMSO (Sigma Aldrich, D2650) in CSM-I (Cell Staining Buffer – Intracellular), consisting of 5 mg/ml BSA and 0.2 mg/ml sodium azide in PBS, vortexed and stored at -20 °C until required. Barcoding and intracellular staining. Cells were thawed, 2 ml of CSM-I was added into each sample, washed once with 4 mL PBS and pelleted. Each cell pellet was resuspended in a unique barcoding aliquot from the Cell-ID 20-plex Pd Barcoding Kit (Fluidigm, 201060) in 1 mL of cold PBS, vortexed and incubated at RT for 15 min. The mixtures were diluted in 3 mL of CSM-I, pelleted and washed with CSM-I. Each cell pellet was resuspended in 200 µL of 1x FOXP3 Permeabilization Buffer each, pooled into a 5 mL polypropylene FACS tube and pelleted. For each sample included in the pooled sample, 10 µL of 100 U/mL heparin sodium salt in PBS and 0.5 µL of Fc block was added and the sample gently mixed. Intracellular antibody staining. After incubating for 5 min at RT in the dark, a master mix of intracellular targeting, metal-conjugated antibodies (Supplementary Table 2) in CSM-I was added. For each sample included in the pooled sample, one equivalent of antibody and 25 µL of CSM-I was used. The sample was gently mixed and incubated on ice in the dark for 45 min. Cells were washed twice CSM-I. The cell pellet was resuspended in 1 mL of PBS and vortexed well. For every individual sample included in the pooled sample, a minimum of 500 µL of 4% Paraformaldehyde (PFA) (Thermo Fisher, 28908) in PBS was added to fix the cells. The fixed sample was vortexed and stored overnight at 4 °C in the dark until the mass cytometry analysis following day.

Mass cytometry DNA staining and acquisition
On the day of acquisition, 5 µL of 125 µM of Cell-ID Iridium Intercalator (Fluidigm, 201192A) was added to the cells and vortexed well. After 1 h of incubation at RT, the cells were washed once with PBS, twice in water and resuspended at a concentration of 1x106 cells/mL in 15% EQ Four Element Calibration Beads (Fluidigm, 201078) in water. This was filtered twice through a 70 µm cell strainer and acquired on a Helios Mass Cytometer (Fluidigm), using a Super Sampler (Victorian Airship & Scientific Apparatus LLC).

Mass cytometry data processing
FCS files were normalised for signal-drift during the acquisition run using the in-built Helios normalisation tool (Fluidigm). For in vitro fibroblast analysis, individual sample events were deconvoluted using manual gating based on the pre-determined ITGB1 barcode after removing non-fibroblast cell types based on expression of canonical markers and GFP. For immune cell analysis of mouse tumours, individual sample events were deconvoluted using the a stand-alone debarcoder 9, with a Mahalanobis distance of 15 and a minimum barcode separation of 0.3. Individual sample FCS files were uploaded to the cloud-based cytometry platform Cytobank (https://www.cytobank.org, Beckmann Coulter) and checked for consistent signal across the entire acquisition period and correct deconvolution. As per standard methods, live cell events were selected based on DNA-191Ir positivity and cisplatin-198Pt negativity. 191Ir+ debris, remaining non-fibroblast cell types, cell doublets and aggregates were removed. M/N/B and T cells were selected by biaxial gating as CD45+ CD3- and CD45+ CD3+, respectively, and exported from Cytobank as FCS files, which were subsequently loaded on Cytofkit2 (version 2.0.1). To analyse the different cell populations in an unbiased way, cells were clustered using FlowSOM 10 and visualised using UMAP projections 11, exporting cell data with annotated clusters for further downstream analysis. Clusters were assigned to specific immune cell populations based on the expression of respective markers, and clusters belonging to the same cell population were merged.

Instrument	BD FACSAria III Cell Sorter (CAF cell sorting)<br>Fludigm Helios (mass cytometry)
Software	BD FACSDiva Software<br>Cytobank<br>Cytofkit2
Cell population abundance	The abundance of FACS-sorted cells was determined by AriaII Diva software, and >98% purity was confirmed.

Gating strategy

For all experiments, debris was excluded based on FSC-A and SSC-A. Single cells were gated based on FSC-W and FSC-A. Live, single cells were gated based on Live/Dead NIR staining. For CAF isolation, CAFs were gated based on Lineage- (CD45, CD31, EpCAM) PDPN+ CD90+ staining.

☒ Tick this box to confirm that a figure exemplifying the gating strategy is provided in the Supplementary Information.
